# Consumer wearable devices for evaluation of heart rate control using digoxin versus beta-blockers: the RATE-AF randomized trial

**DOI:** 10.1038/s41591-024-03094-4

**Published:** 2024-07-15

**Authors:** Simrat K. Gill, Andrey Barsky, Xin Guan, Karina V. Bunting, Andreas Karwath, Otilia Tica, Mary Stanbury, Sandra Haynes, Amos Folarin, Richard Dobson, Julia Kurps, Folkert W. Asselbergs, Diederick E. Grobbee, A. John Camm, Marinus J. C. Eijkemans, Georgios V. Gkoutos, Dipak Kotecha, BigData@Heart Consortium, the cardAIc group, the RATE-AF trial team

**Affiliations:** 1 Institute of Cardiovascular Sciences, University of Birmingham, Birmingham, UK; 2 Institute of Cancer and Genomic Sciences, University of Birmingham, Birmingham, UK; 3 West Midlands NHS Secure Data Environment, University Hospitals Birmingham NHS Foundation Trust, Birmingham, UK; 4 NIHR Birmingham Biomedical Research Centre, University Hospitals Birmingham NHS Foundation Trust, Birmingham, UK; 5 Patient and Public Involvement Team, Birmingham, UK; 6 Department of Biostatistics & Health Informatics, King’s College London, London, UK; 7 Health Data Research UK, University College London, London, UK; 8 Real World Data team, The Hyve, Utrecht, the Netherlands; 9 Department of Cardiology, University Medical Center Utrecht, Utrecht University, Utrecht, the Netherlands; 10 Amsterdam University Medical Center, Department of Cardiology, University of Amsterdam, Amsterdam, the Netherlands; 11 Julius Center for Health Sciences and Primary Care, University Medical Centre Utrecht, Utrecht, the Netherlands; 12 Cardiology Clinical Academic Group, St George’s University of London, London, UK

**Keywords:** Drug therapy, Atrial fibrillation

## Abstract

Consumer-grade wearable technology has the potential to support clinical research and patient management. Here, we report results from the RATE-AF trial wearables study, which was designed to compare heart rate in older, multimorbid patients with permanent atrial fibrillation and heart failure who were randomized to treatment with either digoxin or beta-blockers. Heart rate (*n* = 143,379,796) and physical activity (*n* = 23,704,307) intervals were obtained from 53 participants (mean age 75.6 years (s.d. 8.4), 40% women) using a wrist-worn wearable linked to a smartphone for 20 weeks. Heart rates in participants treated with digoxin versus beta-blockers were not significantly different (regression coefficient 1.22 (95% confidence interval (CI) −2.82 to 5.27; *P* = 0.55); adjusted 0.66 (95% CI −3.45 to 4.77; *P* = 0.75)). No difference in heart rate was observed between the two groups of patients after accounting for physical activity (*P* = 0.74) or patients with high activity levels (≥30,000 steps per week; *P* = 0.97). Using a convolutional neural network designed to account for missing data, we found that wearable device data could predict New York Heart Association functional class 5 months after baseline assessment similarly to standard clinical measures of electrocardiographic heart rate and 6-minute walk test (F1 score 0.56 (95% CI 0.41 to 0.70) versus 0.55 (95% CI 0.41 to 0.68); *P* = 0.88 for comparison). The results of this study indicate that digoxin and beta-blockers have equivalent effects on heart rate in atrial fibrillation at rest and on exertion, and suggest that dynamic monitoring of individuals with arrhythmia using wearable technology could be an alternative to in-person assessment. ClinicalTrials.gov identifier: NCT02391337.

## Abstract (web summary)

In a substudy of the RATE-AF trial, which compared heart rate control therapy using digoxin or the beta-blocker bisoprolol, heart rate and physical activity data collected using a wearable device showed equivalent heart rate control by the two drugs and could be used to predict future heart failure functional class as well as standard clinical measurements.

## Main

The effectiveness and safety of therapeutic interventions are traditionally evaluated with periodic, single time-point assessments. In patients with cardiovascular disease, this often requires multiple clinical visits to obtain tests such as electrocardiograms (ECG) for assessment of heart rate, or 6-minute walk (6MW) tests to appraise physical capacity. These in-hospital measurements are time-consuming, costly and not dynamic, providing a limited ‘snapshot’ of that person’s functional status^1,2^. Wearable technology can provide continuous measurement of physiological parameters^3–5^. However the large volume of data acquired may need advanced analytics, taking account of the lower quality compared with medical devices or frequent missing values^6^.

A prime example of the potential clinical value of wearable sensor data could be therapy choice and dose adjustment for heart rate control in patients with atrial fibrillation (AF), an increasingly common rhythm disorder. There is a limited evidence base on this topic; for example, digoxin has typically been considered a poorly effective drug for controlling heart rate, particularly on exertion, although this is based on acute studies only^7^. Wearables offer an opportunity to assess each patient in their own environment, with longer-term evaluation better reflecting the extended time taken to achieve therapeutic benefit from digoxin^8^, and properly account for physical activity.

Although there is considerable potential for consumer-wearable devices to contribute to cardiovascular disease management, there are few robust studies independent of manufacturers that can highlight opportunities as well as limitations in older, multimorbid patients^9^. Embedded in a randomized controlled trial to mitigate the effects of unmeasured or unknown confounders^10^, we hypothesized that a wrist-worn wearable could: (1) address whether digoxin is inferior to beta-blockers for longer-term heart rate control in patients with AF at rest and on exertion; (2) adjust for differences in individual physical activity; and (3) explore whether wearable sensor data are comparable with conventional measurements for the prediction of clinical progress, and ultimately, aid longer-term patient management.

## Results

**Fig. 1:**
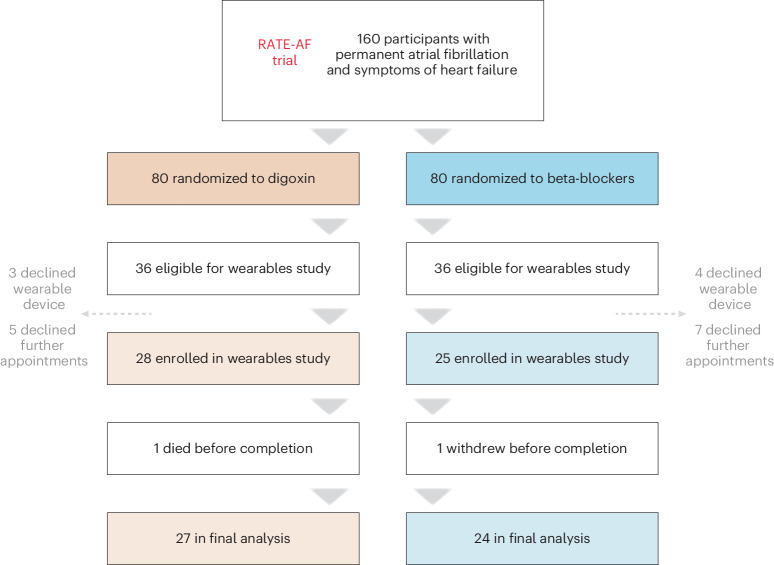
RATE-AF wearables substudy flowchart. Flowchart for the wearables study enrollment.

One hundred and sixty patients were randomized to digoxin or beta-blocker therapy in the RAte control Therapy Evaluation in permanent Atrial Fibrillation (RATE-AF) trial, of whom 72 (36 from each treatment arm) were eligible to participate in a substudy that provided a wrist-worn wearable and a connected smartphone. The design and deployment of the study were aided by a patient and public involvement team^11^. The remaining participants had completed the main trial or did not have sufficient time left for data collection (Fig. 1). Of those eligible, 8 in the digoxin group and 11 in the beta-blocker group declined to participate, principally because they did not want to use a wearable device or attend further trial appointments. The characteristics of those who declined were similar to the participating cohort, apart from more women declining and with a lower 6MW distance (Extended Data Table 1).

**Extended Data Fig. 1:**
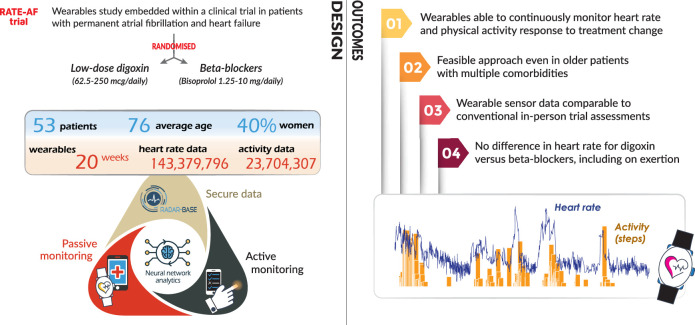
Overview of the RATE-AF randomized trial wearable study. mcg = micrograms; mg = milligrams; RATE-AF = RAte control Therapy Evaluation in permanent Atrial Fibrillation.

Fifty-three participants were enrolled in the substudy (Extended Data Fig. 1), with mean age at randomization of 75.6 years (s.d. 8.4; range 61 to 90 years) and 40% women. Twenty-eight participants (53%) had been randomized to digoxin and 25 (47%) to beta-blockers a mean of 30 weeks before their entry into the wearables substudy (s.d. 8 weeks; range 12 to 46 weeks). Both groups were well balanced with respect to demographics and clinical measurements (Table 1), with the most common comorbidities being hypertension (74%) and heart failure (45%). There were more patients with a formal diagnosis of heart failure in the digoxin group, but ECG-derived left-ventricular ejection fraction (LVEF) measured in a blinded fashion at the start of the trial was similar for both groups: 55.7% (s.d. 8.8%) for digoxin versus 55.4% (s.d. 9.0%) for beta-blockers. One patient from each arm was lost to follow-up.

**Table 1:** Baseline characteristics

Characteristic	Overall (*N* = 53)	Randomized to digoxin (*N* = 28)	Randomized to beta-blockers (*N* = 25)
**Demographics**			
Age at randomization, mean years (s.d.)	75.6 (8.4)	74.2 (8.4)	77.2. (8.3)
Gender, women *n* (%)	21 (39.6)	11 (39.3)	10 (40.0)
**Baseline comorbidities**			
Hypertension, *n* (%)	39 (73.6)	22 (78.6)	17 (68.0)
Diabetes mellitus, *n* (%)	11 (20.8)	5 (17.9)	6 (24.0)
Previous stroke or transient ischemic attack, *n* (%)	8 (15.1)	5 (17.9)	3 (12.0)
Treatment with inhalers for COPD or asthma, *n* (%)	14 (26.4)	8 (28.6)	6 (24.0)
Diagnosed with heart failure, *n* (%)	24 (45.3)	16 (57.1)	8 (32.0)
LVEF on echocardiogram, mean % (s.d.)	55.6 (8.8)	55.7 (8.8)	55.4 (9.0)
Echocardiogram LVEF <50%, *n* (%)	18 (34.0)	11 (39.3)	7 (28.0)
**Clinical measurements at baseline trial visit**			
ECG heart rate, mean bpm (s.d.)	97.7 (20.4)	95.7 (19.8)	99.6 (20.5)
Systolic blood pressure, mean mmHg (s.d.)	137.9 (17.2)	136.2 (15.2)	139.7 (19.3)
6MW distance, median meters (i.q.r.)	384 (207–437)	372 (150–434.5)	384 (229–438)
NT-proBNP, median pg ml^−1^ (i.q.r.)	1099 (770–1725)	1112 (766–1831)	1057 (829–1717)
NYHA class, *n* (%)			
I (no limitation of activity)	0 (0)	0 (0)	0 (0)
II (slight limitation of activity)	38 (71.7)	22 (78.6)	16 (64.0)
III (marked limitation of activity)	14 (26.4)	5 (17.9)	9 (36.0)
IV (symptoms of heart failure at rest)	1 (1.9)	1 (3.6)	0 (0)
mEHRA class, *n* (%)			
1 (no symptoms)	0 (0)	0 (0)	0 (0)
2a (mild symptoms)	3 (5.7)	1 (3.6)	2 (8.0)
2b (moderate symptoms)	26 (49.1)	16 (57.1)	10 (40.0)
3 (severe symptoms)	22 (41.5)	11 (39.3)	11 (44.0)
4 (disabling symptoms)	2 (3.8)	0 (0)	2 (8.0)

COPD, chronic obstructive pulmonary disease.

Heart rate at the start of the substudy was 79.4 beats per minute (bpm; s.d. 12.2) in those randomized to digoxin and 73.1 bpm (s.d. 14.3) for beta-blockers, with one patient in each group presenting at that visit in sinus rhythm. The capacity for physical activity was similar in both groups at baseline, with a median 6MW distance of 351 m for digoxin (i.q.r. 120–454) and 357 m for beta-blockers (i.q.r. 295–411). Symptoms of AF and heart failure were reported in all patients (100%), with modified European Heart Rhythm Association (mEHRA) class 2a or above, and New York Heart Association (NYHA) class II or above. At the trial mid-point, the mean dose of digoxin used was 157 µg (s.d. 10 µg) with a serum digoxin level of 0.79 ng ml^−1^ (s.d. 0.29); the mean dose in the beta-blocker group was 3.2 mg of bisoprolol (s.d. 1.9). The duration of the wearable substudy was a median of 23 weeks (i.q.r. 4–38). There was consistent use of the wrist-worn wearable (more so than the smartphone), with 90.4% of participants using this every day for the last 7 days before the interim review (Extended Data Table 2).

### Wearable-acquired heart rate and physical activity

**Fig. 2:**
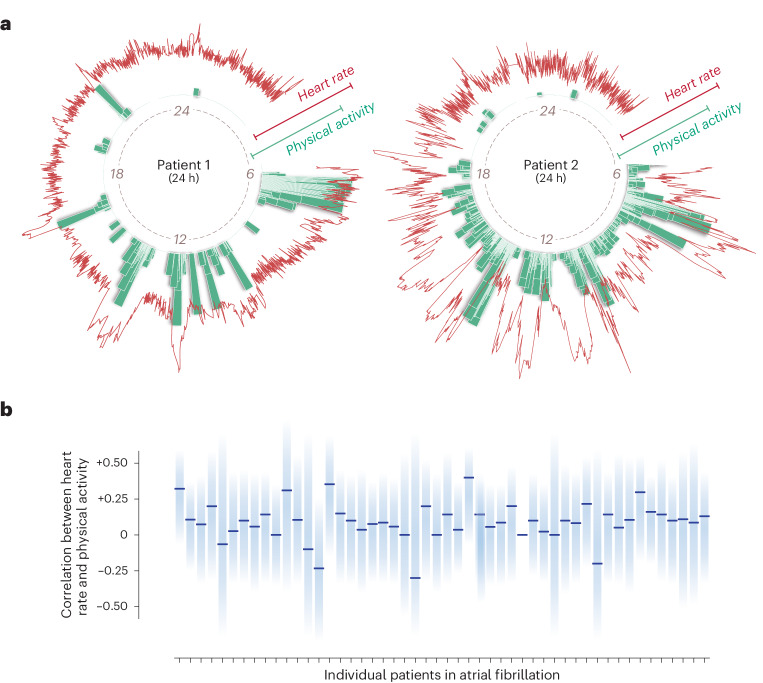
Wearable device data for measurement of heart rate and physical activity. **a**, Examples of data capture for heart rate (red lines) and step count (green bars) using a wrist-worn wearable and smartphone over a single 24-h period for two individual patients with AF and heart failure. **b**, Correlations between daytime 10-s intervals of heart rate and physical activity for 50 patients who remained in AF at each visit. Light blue columns indicate the range of positive and negative correlations between heart rate and physical activity, with medians indicated by dark blue bars (correlation <0.19, very weak; 0.20–0.59, weak to moderate).

Per patient, the mean duration of ambulatory sensor data collected was 20 weeks (s.d. 7), with an average of 2,623,951 heart rate data points for each patient treated with digoxin (s.d. 907,697) and 2,796,367 for each patient treated with beta-blocker (s.d. 811,956). Across all patients there were 143,379,796 data intervals collected for heart rate and 23,704,307 for corresponding physical activity (Table 2). Figure 2 highlights the considerable variability within and across individual patients despite appropriate rate control therapy in terms of heart rate, response to exertion and the correlation between heart rate and physical activity.

**Table 2:** Sensor data from the wearable device

Data collection	Randomized to digoxin (*N* = 28)	Randomized to beta-blocker (*N* = 25)
Total number of data points for heart rate	73,470,613	69,909,183
Total number of data points for step count	12,210,254	11,494,053
Number of combined data points for heart rate and step count	4,746,169	4,683,959
Mean number of data points for heart rate per patient (s.d.)	2,623,951 (907,697)	2,796,367 (811,956)
Mean number of data points for step count per patient (s.d.)	436,081 (122,493)	459,762 (110,443)
Mean number of combined data points for heart rate and step count per patient (s.d.)	169,506 (71,217)	187,358 (57,784)
Mean timespan for data collection per patient, days (s.d.)	153 (53)	160 (46)

The data presented are for nonmissing time points.

### Digoxin versus beta-blockers

**Fig. 3:**
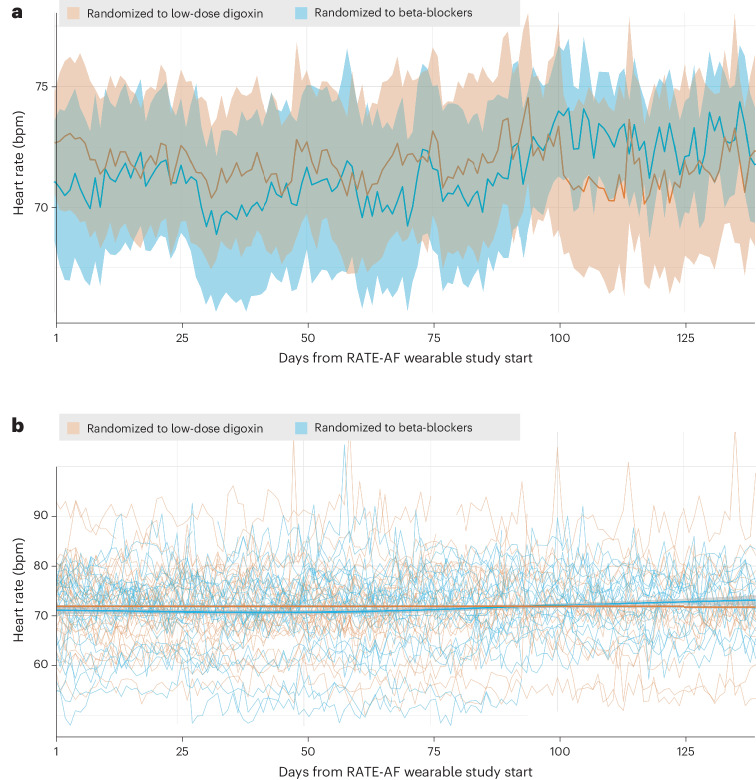
Heart rate data from the wearable device. The start of the wearables study was preceded by a period of dose adjustment and stabilization (mean of 30 weeks from randomization). **a**, Mean (solid line) and s.d. (shaded area) in heart rate over the 20-week period of follow-up in patients randomized to treatment with digoxin (brown) or beta-blockers (blue). **b**, Individual patient heart rate trajectories over the 20-week period of follow-up in patients randomized to treatment with digoxin (brown) and beta-blockers (blue). Bold lines indicate the fitted generalized linear model curves with corresponding 95% CI (shaded). No significant difference was demonstrated between the two groups using a generalized linear model with random-effects to account for repeated measurements (unadjusted *P* = 0.55; adjusted *P* = 0.75; after accounting for physical activity *P* = 0.74).

Weekly averages of heart rate were no different when comparing patients randomized to digoxin or beta-blockers (Extended Data Table 3). Accounting for all repeated measurements over time, there was no significant difference in heart rate comparing digoxin and beta-blocker therapy using the wearable sensors (Fig. 3). There was no interaction seen according to gender (*P*_interaction_ = 0.39). The unadjusted regression coefficient for digoxin versus beta-blockers was 1.22 (95% CI −2.82 to 5.27; *P* = 0.55), and 0.66 when adjusted for age, gender, diagnosis of heart failure and N-terminal pro-hormone B-natriuretic peptide (NT-proBNP) (95% CI −3.45 to 4.77; *P* = 0.75). There remained no difference in heart rate between the digoxin and beta-blocker groups after accounting for physical activity (*P* = 0.74). Post-hoc adjusted subgroup analysis according to activity levels found no difference in heart rate between digoxin and beta-blockers in those with low weekly-averaged activity (<15,000 steps per week; 298 weeks from 44 patients; *P* = 0.48), minimum recommended activity (15,000–30,000 steps per week; 316 weeks from 37 patients; *P* = 0.47) or recommended activity (≥30,000 steps per week; 417 weeks from 33 patients; *P* = 0.97).

### Exploratory analysis of wearable data to predict NYHA class

**Fig. 4:**
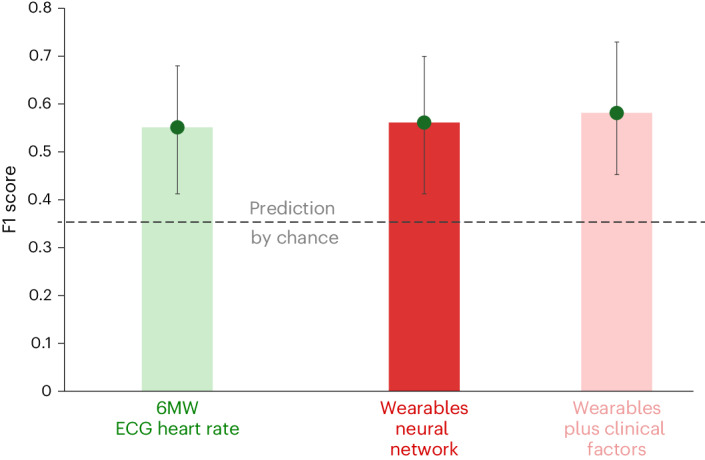
Prediction of clinical progress with a neural network based on wearable data. F1 scores combining precision and recall of each model are presented along with 95% CI for the prediction of NYHA functional class at the end of follow-up (mean 5 months); an F1 score of 0.35 (dashed line) is equivalent to chance. Derived from wearable sensor data from *n* = 41 individual patients.

A convolutional neural network (CNN) model was trained on heart rate and step count data using the wearable sensor output from 41 patients who had sufficient time windows for analysis. F1 scores (combining precision and recall) were used to compare the CNN model for prediction of NYHA class at the end of the trial (Extended Data Table 4), with chance returning an F1 score of 0.35. The wearables CNN yielded an F1 score of 0.56 (95% CI 0.41 to 0.70); Fig. 4. This was equivalent to a model generated from conventional trial parameters (ECG heart rate and 6MW test results), which returned an F1 score of 0.55 (95% CI 0.41 to 0.68); *P* = 0.88 for patient-level comparison with wearables CNN. The wearable data appeared independent of clinical factors such as age, gender and body mass index, with similar F1 score when combining wearable data with clinical factors (0.58; 95% CI 0.45 to 0.73). The corresponding areas under the receiver operator characteristic curves were 0.73 for ECG heart rate and 6MW test, 0.77 for the wearables CNN and 0.78 for wearables plus clinical factors.

## Discussion

The RATE-AF wearables study demonstrated a potential use of nonmedical-grade wearable devices in clinical research where they were used to monitor clinical progress and the response to a change in therapy. Embedded in a randomized trial, the study provides robust information on the value, but also the limitations of using these devices. The commonly held view originating from acute trials that digoxin is inferior to beta-blockers for heart rate control in AF was not seen in this longer-term study. No difference in heart rate was seen when considering more than 140 million data points at rest and on exertion during a 20-week period. The equivalence of digoxin to beta-blockers held after accounting for individual differences in day-to-day physical activity, and no difference in heart rate was evident even in periods with high activity. Although limited by the number of patients, the wearable sensor data appeared to be comparable with conventional trial outcomes for prediction of a clinical outcome (in this case, functional class), but required the development of a neural network pipeline for appropriate analytics. Standardization of these approaches could lead to wearable devices contributing to, or even replacing resource-intensive clinical tests and visits in the future.

The majority of clinical trials in cardiovascular research utilize in-person periodic testing that not only requires patient and staff attendance, but is time-constrained in an environment unlike the patient’s own surroundings. Wearables offer an exciting possibility for patient-directed data collection that better reflects real-life day-to-day variations in heart rate and physical activity^12^. In this study, a neural network that was self-training was designed to provide value from the vast amount of information collected by wearables, and address key issues such as inconsistent and missing data. The performance of the wearable neural network was not dependent on clinical factors, and for prediction of future NYHA class was equivalent to (but not better than) conventional parameters such as a 12-lead ECG and 6MW testing. F1 scores >0.5 indicate an acceptable balance between precision and recall, and the wearable model score of 0.56 was significantly better than chance (in this case, 0.35). Further development, testing and validation of these approaches is clearly required. Of note, the trial recruited older patients (mean age 76 years, range up to 90 years) who indicated the use of wearables was appealing to monitor and improve their own functional capacity.

Several studies have utilized wearable devices in cardiovascular research. The Apple smartwatch and Fitbit studies used photoplethysmography to identify new AF^13,14^, and there are many different devices being used across heart failure to quantify patient physiology^15^. Early optimism has already started to fade somewhat, and one retrospective matched study in the US found that patients using wearable devices accessed more healthcare but with no difference in heart rate^16^. It remains to be seen how these consumer-bought, nonmedical devices will be integrated into routine care, with or without the acceptance of healthcare professionals^1,17^. Further randomized trials are needed to understand whether continuous monitoring can provide a personalized assessment of treatment response, or help to identify subphenotypes of disease. Similarly, approaches to transparent and validated artificial intelligence (AI) remain in their infancy^18^. AI has demonstrated an ability to go beyond our current linear understanding of disease trajectory and interactions, with the ability to personalize diagnostic and therapeutic strategies even in multimorbid conditions^19,20^. Despite the potential benefits of AI, broader use in clinical practice requires an approach guided by strong methodological principles^6^.

AF is a key public health issue, predicted to double in prevalence over the next few decades^21^. Recent evidence suggests that cognitive decline and vascular dementia should be added to the list of adverse events suffered by patients with AF^22,23^. New technologies have rapidly increased our ability to detect AF, but how these should be implemented in clinical practice remains unclear^9^. Despite permanent AF being the most common ‘type’ of AF^24^, there is a remarkable lack of research to support decision-making and improve patient quality of life^7,25–27^. Treatment choices for heart rate control are often made based on evidence from heart failure trials; however, the reduction in mortality from beta-blockers was not evident in double-blind trials in patients with heart failure and concomitant AF^28^. Digoxin has traditionally been reserved for sicker patients or as a second-line agent. Similar to beta-blockers, there is no apparent mortality impact from digoxin in patients with AF and heart failure, and observational analyses are wholly inappropriate to evaluate outcomes with digoxin because of systematic prescription biases^29,30^. The RATE-AF trial was a head-to-head randomized trial of digoxin and beta-blockers, showing that digoxin had a similar impact on patient-report quality life as beta-blockers yet substantially improved functional class. Use of digoxin led to a significant reduction in natriuretic peptides, and fewer than half the number of primary care and unplanned hospital visits^10^. This study now adds a further dimension, showing that low-dose digoxin can be an effective rate control agent. The effect on heart rate and physical activity was consistent with the longer-term activity of digoxin, including pro-parasympathetic effects on cellular, electrophysiological and neurohormonal pathways^8^.

Although the number of patients was limited and did not encompass all participants in RATE-AF, this study included a large volume of wearable data and was able to benefit from being embedded in a randomized trial to limit extraneous bias. The wearables were implemented post randomization and hence there is a risk of residual confounding; however, there was no crossover use of digoxin^10^, analysis was by intention-to-treat and reasonable balance in clinical characteristics between groups was maintained for those patients joining the wearable study. As expected, actual usage of wearable devices among participants had considerable variation. There is a known motivational and behavioral impact from wearables, although when asked at final follow-up, the same proportion in each randomized group indicated improved motivation for physical activity: 16 patients (59.3%) in the digoxin group and 13 (54.2%) in the beta-blocker group (*P* for comparison = 0.71). Use in an older population brought challenges, with many participants not previously owning a smartphone; however, good compliance and data quality were achieved. Missing data points were frequent, but their impact was minimized by using an innovative approach to embed machine learning on the significance of missing data, rather than ignore or impute it. Although subgroup analysis according to activity levels found no difference in heart rate between digoxin and beta-blockers, the use of digoxin in high-activity settings remains largely untested. Diversity in ethnicity was limited (white British or Irish ethnicity accounted for 94% of participants), and so these results cannot be extrapolated to other ethnicity groups. Although technology advances can improve human health, it remains important to ensure that deployment of such devices does not exacerbate health inequalities.

In summary, a wrist-worn consumer-grade wearable device and smartphone were successfully deployed within a randomized controlled trial of older, multimorbid patients to evaluate continuous, ambulatory heart rate and physical activity. Including an average of two to three million data points per patient, including at rest and exertion, digoxin and beta-blocker therapy had similar effects on heart rate measured over a 20-week period. A neural network model of wearable sensor data showed similar performance for predicting future health status as conventional measures used in clinical trials.

## Methods

### The RATE-AF trial

The RATE-AF trial was a prospective, randomized, open-label, blinded end-point trial that compared the use of low-dose digoxin versus beta-blockers for long-term heart rate control^7^. Recruitment took place across primary care sites and three hospitals in the West Midlands region of England between 2016 and 2018. Inclusion criteria were: (1) age 60 years or older; (2) permanent AF in need of rate control; and (3) symptoms of heart failure, with breathlessness equivalent to NYHA class II or above. Exclusion criteria were limited so that the trial population reflected routine clinical practice (see published protocol for full list of selection criteria)^10^.

### Ethics and inclusion statement

The trial was co-designed by a patient and public involvement (PPI) team, with the aim of improving quality of life for patients with AF^11,27^. Ethical approval was obtained from the East Midlands–Derby Research Ethics Committee (16/EM/0178), the Health Research Authority (IRAS 191437) and the Medicines and Healthcare Products Regulatory Agency. The trial was publicly funded by the UK National Institute for Health and Care Research (CDF-2015-08-074) and registered with clinicaltrials.gov (NCT02391337) and clinicaltrialsregister.eu (2015-005043-13).

### Randomization and trial process

Each participant was randomized to either digoxin 62.5–250 µg or bisoprolol 1.25–10 mg once daily in a 1:1 ratio at their baseline visit. Randomization was completed using a computer-generated minimization algorithm to ensure treatment arms were balanced for gender and AF symptoms, based on the mEHRA classification.

The trial was embedded into usual care within the National Health Service (NHS), with participants attending formal visits at baseline, 6 months and 12 months. Endpoints acquired were patient-reported quality of life, NT-proBNP, symptoms and functional capacity using mEHRA and NYHA class, 6MW distance and time, heart rate (pulse examination), 12-lead ECG, LVEF using cardiac ultrasound and assessment of adverse events.

### Wearables substudy

Funding for the wearables substudy was obtained after the main trial had commenced from the European Union Innovative Medicines Initiative BigData@Heart program (grant no. 116074). The study was supported by the Application of Artificial Intelligence to Routine Healthcare Data to Benefit Patients with Cardiovascular Disease (card*AI*c) team at the University of Birmingham and University Hospitals Birmingham NHS Foundation Trust. An amendment was made to the trial protocol and subsequently approved by the Research Ethics Committee. One of the original stated aims of the substudy was to correlate wearable sensor data with patient quality of life using the Short Form (36) Health Survey (SF-36). Following work led by the PPI team that showed SF-36 to be as suboptimal measure of assessment^27^, this was subsequently changed to NYHA class in the statistical analysis plan completed before data analysis (Supplementary Note).

Participants with at least 2 months remaining in the RATE-AF trial were considered eligible for inclusion in the substudy. All participants were provided with a specific patient information leaflet written by the PPI team, and were asked to sign an optional form to indicate informed consent. As an exploratory analysis, no sample size calculation was performed in advance of recruitment. Heart rate sensor data from the first 5 weeks in the first ten participants was used to estimate the minimum number of participants needed. The average weekly heart rate, s.d. and correlation of repeated measures from this data indicated that a sample size of 40 participants would provide 90% power over 20 weeks to detect a 1/3 s.d. difference in heart rate (2 bpm) between digoxin and beta-blockers (control 72 bpm; s.d. 6 bpm; repeated measures correlation 0.91; two-sided alpha 0.05). A minimum target of 50 enrolled participants would account for death and loss to follow-up during the substudy.

Consenting individuals were given a Samsung A6 Android smartphone (with a prepaid mobile data contract) and wrist-worn Fitbit Charge 2 wearable device for passive monitoring. There were no exclusions related to age or previous proficiency with information technology. Applications were preinstalled and set up for remote data collection, providing active monitoring and an educational resource for patients, including the European Society of Cardiology smartphone application (app) specifically designed for patients with AF^31^. Participants were shown how to charge and carry out basic functions on each device, and how to use the apps. They were instructed to carry the phone throughout the day and to wear the wrist device continuously, only removing it for showering, bathing, swimming or charging. After the set-up appointment, in-person or telephone follow-up was provided after the first week, after 4 weeks and ad hoc to maintain engagement and address any concerns or technical issues raised by participants.

### Data collection and storage

Data collected via the device and smartphone was encrypted and uploaded to a secure server, temporarily cached on the smartphone until an appropriate Wi-Fi or mobile data connection was available. The collection of wearable data streams was automated using the RADAR-base platform, funded by the European Union Innovative Medicines Initiative RADAR-CNS (grant no. 115902)^32^. This platform allows for secure streaming of data from wearables, apps and devices to a central location. For this study, the RADAR-base platform was installed on a virtual machine hosted by Amazon Web Services in the Europe (London) region and was maintained by the Hyve (IT company, Netherlands). By applying for Fitbit developer application, the RADAR-base platform automatically collected data from registered participants, who were also able to see their own heart rate and step counts. For clinical data storage, secure electronic case report forms were generated using the Research Electronic Data Capture (REDCap) system hosted by the University of Birmingham, and the main trial case report forms hosted by the Birmingham Clinical Trials Unit.

### Statistical analysis

Data were analyzed by intention-to-treat according to the randomized allocation (digoxin versus beta-blockers), with no imputation for any missing data. Continuous measurements of heart rate and step count were pooled at 1-min intervals to form time-series data (heart rate averaged over each minute; step counts summed over each minute), with the primary analysis over a prespecified period of the first 20 weeks of device use. The results were summarized and presented as a number, percentage, mean and s.d. or standard error of the mean, or median with i.q.r. The Kruskal–Wallis nonparametric test or a *t*-test were used to determine differences between the two treatment arms depending on normality, and Spearman’s test was used to quantify correlations. To account for multiple repeated measurements of heart rate over time in individual participants, generalized linear models were generated using a random-effects estimator and exchangeable correlation matrix. A post-hoc subgroup analysis according to activity levels was based on US Centers for Disease Control recommended activity levels (minimum 150 min per week aerobic activity equivalent to 15,000 steps per week, and health benefits goal of 300 min per week aerobic activity equivalent to 30,000 steps per week). Statistical analyses were performed using Stata v.17 (StataCorp LP), with a two-tailed *P*-value <0.05 denoting statistical significance.

### Neural network

**Extended Data Fig. 2:**
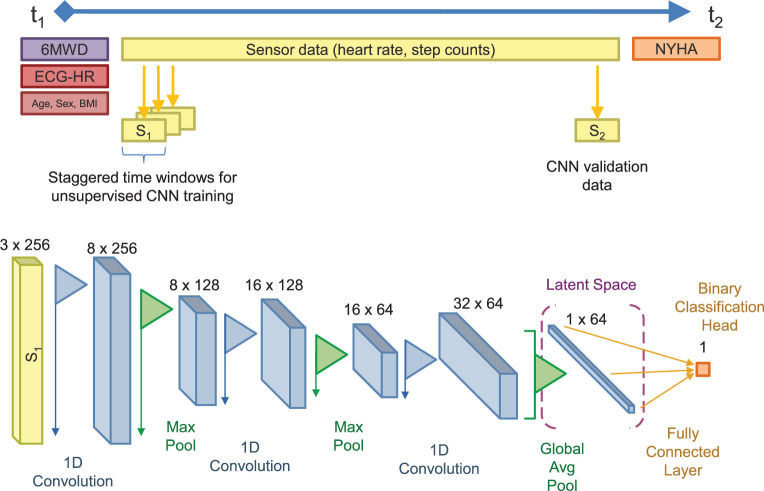
Neural network architecture. Top panel: At time-point 1 (t1), 6-minute walk distance (6MWD), electrocardiogram (ECG) heart rate (HR), age, sex and body mass index are taken at the closest trial appointment to the start of the wearables sub-study. S1 denotes start of sensor data collection. S2 denotes sensor data used for validation. Time-point 2 (t2) is the future for New York Heart Association (NYHA) functional class prediction. Lower panel: The convolutional neural network (CNN) architecture uses dilated convolution and max-pool layers to capture long-term structure. Global average pooling is used to reduce spatial extent to fixed-length vector representation. Dropout and L2 penalty are applied to avoid overfitting to training set and label smoothing is used for faster learning. The final layer is binary sigmoid for data discrimination task. The penultimate layer of 32 units is used as latent space features for regression modeling.

Machine learning algorithms were generated to explore whether continuous sensor data were comparable with conventional periodic trial measurements at the closest RATE-AF trial appointment, developed according to our previously published AI framework^6^. Unlabeled wearable sensor data from staggered 4-h periods were used to develop a self-supervising CNN (Extended Data Fig. 2). The self-supervising model was motivated by the principle that important information is carried not only in the heart rate and step count channels, but also in the temporal interaction between those channels. To learn this interaction, an auxiliary dataset was synthesized from a training set of the original sensor data where the heart rates and step counts of each sample were scrambled across patients and dissociated. For example, a multichannel sample might include the heart rate time-series of patient A, but the step count time-series of patient B. The auxiliary dataset was combined with the original data to create a classification problem: to discriminate whether a given sample came from the original or scrambled data. Because the original data have temporal interdependencies between the channels, and the scrambled data do not, it is believed that learning this objective is equivalent to learning the relationship between those sensor channels.

Heart rate measures were standardized to *z*-scores; step counts were normalized to the range [0,1] because of frequent and meaningful measurements of zero (inactivity). Both were defined with respect to each participant’s individual statistics—a heart rate *z*-score of 0 indicates the mean average heart rate for that patient. Small amounts of missing data were present throughout the recordings: these may have been short periods in which participants were not wearing their devices, or where data was not received because of connectivity issues. This missing data was neither dropped nor imputed, but used as a third time-series channel alongside heart rate and step count. This allowed the model to learn the significance of missing data instead of making assumptions about its distribution.

Multichannel time-series data were the input for a one-dimensional convolutional layer of 8 filters and a kernel size of 21 (minutes), followed by a one-dimensional max pooling layer of size and stride 2. After pooling are two further convolutional layers with 20 and 32 filters, each with kernel size 21. Finally, one-dimensional global average pooling was performed to reduce the data representation to a vector of length 32. During training, dropout (with probability = 0.5) was applied to this layer to improve regularization. Finally, the prediction is made by a fully connected layer comprising a single sigmoid unit. Every layer but the last used rectified linear unit activation, and the network was trained to minimize binary cross-entropy using an Adam optimizer with a learning rate of 3 × 10^−4^ and L2 regularization with weight 1 × 10^−8^ applied to each nonbias parameter. After training the network to convergence, the dropout and classification layers were removed from the model, and the output of the final convolutional layer was used as a 32-dimensional embedding vector representing the time-series data used as input.

Because the objective of this model was to predict the patient’s future NYHA class, it was evaluated by embedding data from each patient’s first week, and using that embedding to predict their NYHA class at the end of trial as the outcome of interest. This self-supervised model was trained using all data for each patient other than in this first week, while also holding out a subset of patients as a validation set to monitor under- or overfitting during model training. The hold-out set comprised 20% of the patient group, repeated across five iterations with *k*-fold cross-validation. For this exploratory analysis, participants were only included if they had available nonmissing time windows in the first and subsequent 19 weeks, and reached the final follow-up assessment for NYHA class. Models were compared for prediction of NYHA class at the end of the trial (5 months later): a conventional logistic regression model including ECG heart rate and 6MW test results (distance traveled, time taken and participant speed), and the wearable sensor model using CNN latent time-series embeddings from wearable sensor data as input features with L2 norm regularization (as used in ridge regression).

We prespecified evaluation of models using the F1 score—a metric combining precision and recall that ranges from 0 to 1, with 1 indicating perfect accuracy for classification. For each model, label smoothing was used over the NYHA class targets as a further method of regularization. The 95% CI was estimated by bootstrap resampling. During the peer review process, a post-hoc analysis was added to calculate the area under the receiver operator characteristic curve for each model, which provides an aggregate measure of classification performance with values ranging from 0 to 1 (higher indicates better performance). Machine learning analyses were performed using Python (Python Software Foundation) with scikit-learn, and TensorFlow (Google Brain).

### Role of the funding sources

None of the organizations providing funding had any role in the design or conduct of the study (including collection, analysis and interpretation of the data) or any involvement in preparation, review or approval of the manuscript.

### Reporting frameworks

The study is reported according to the Minimum Information about Clinical Artificial Intelligence Modeling (MI-CLAIM) checklist^33^.

### Reporting summary

Further information on research design is available in the Nature Portfolio Reporting Summary linked to this article.

## Online content

Any methods, additional references, Nature Portfolio reporting summaries, source data, extended data, supplementary information, acknowledgements, peer review information; details of author contributions and competing interests; and statements of data and code availability are available at 10.1038/s41591-024-03094-4.

### Supplementary information

Supplementary Information Statistical and AI analysis plan addendum.

Reporting Summary

## Figures and Tables

**Extended Data Table 1:**
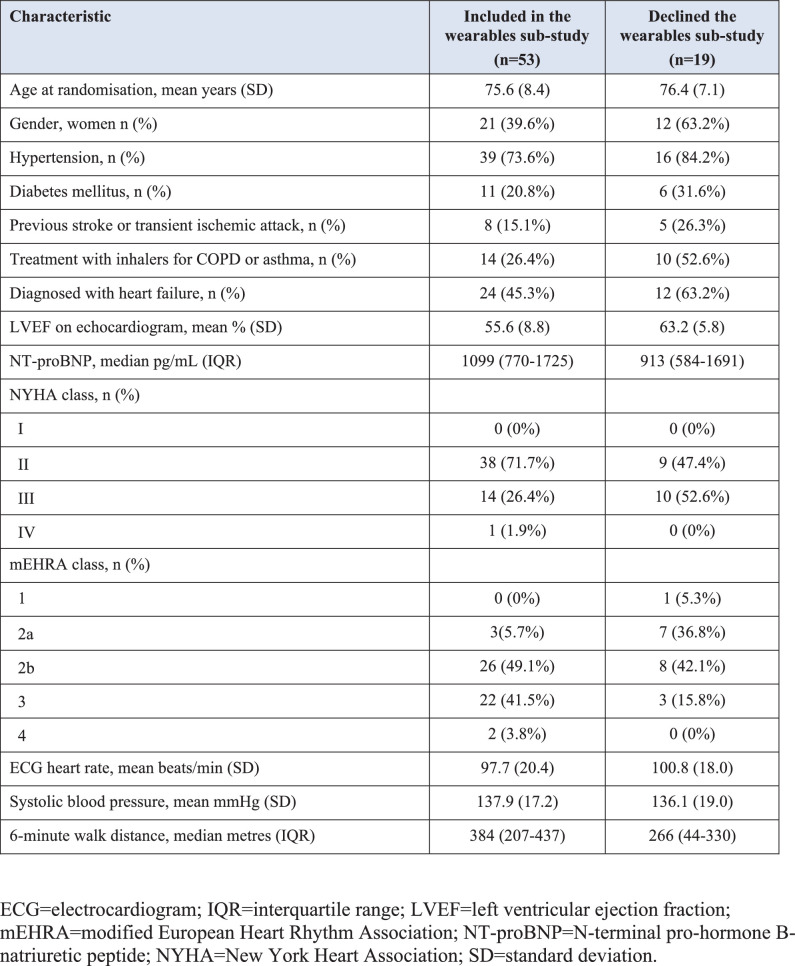
Baseline characteristics of patients that declined participation

**Extended Data Table 2:**
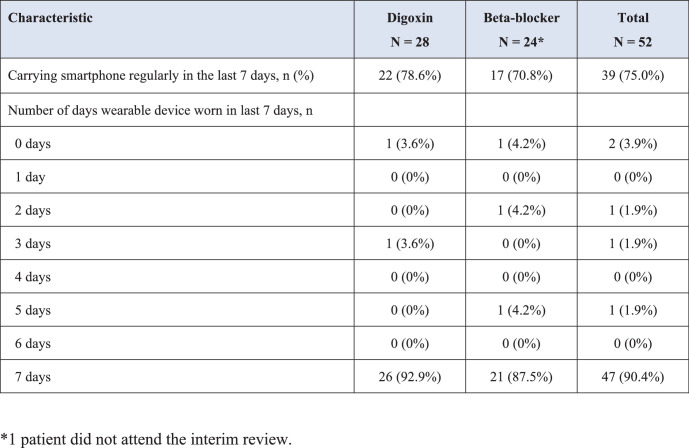
Usage of devices at interim review

**Extended Data Table 3:**
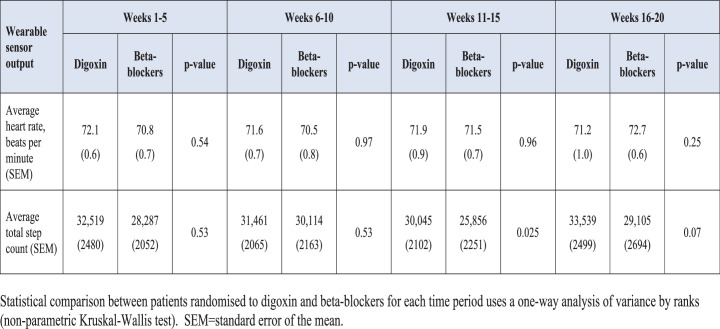
Wearable sensor parameters over sequential weeks

**Extended Data Table 4:**
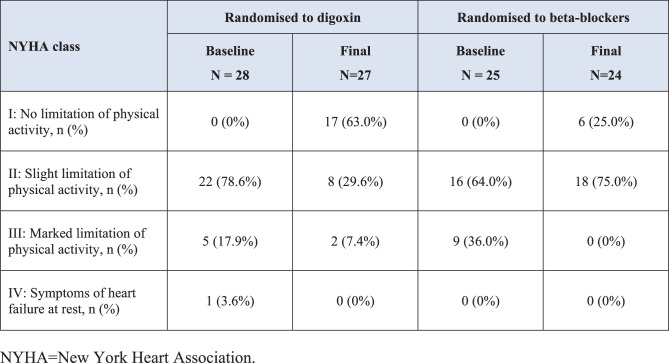
Change in NYHA classification

## Data Availability

Summary anonymized wearable sensor data are available for noncommercial purposes on request to the corresponding author (d.kotecha@bham.ac.uk; 60 days response time for decisions). Because of the risk of patient reidentification, access to any individual-level data will require an appropriate ethical committee approval and review by the RATE-AF trial oversight committee, which includes patient and public representatives (applications to D. Kotecha, d.kotecha@bham.ac.uk; 180 days response time for decisions). Anonymized RATE-AF main trial and substudy datasets will be made available in an open-access repository after completion of secondary manuscripts.

## Code availability

The machine learning framework for embedding multichannel time-series data is made freely available at: https://github.com/gkoutos-group/wearable_data_embedding.
