# Supplementary material for: Consumer wearable devices for evaluation of heart rate control using digoxin versus beta-blockers: the RATE-AF randomized trial
Source: Nat Med. 2024 Jul 15;30(7):2030–6. doi: 10.1038/s41591-024-03094-4 (PMC11271403; doi:10.1038/s41591-024-03094-4)
Supplement: Supplementary file 1 — Statistical and AI analysis plan addendum. [file 41591_2024_3094_MOESM1_ESM.pdf]

nature medicine

Supplementary information https://doi.org/10.1038/s41591-024-03094-4

Consumer wearable devices for evaluation of heart rate control using digoxin versus beta-blockers: the RATE-AF randomized trial

In the format provided by the authors and unedited

UNIVERSITY OF BIRMINGHAM | BCTU Birmingham Clinical Trials Unit

Evaluating different rate control therapies in permanent atrial fibrillation:

A Prospective, randomised, open-label, blinded endpoint trial of comparing digoxin and beta-blockers as initial control therapy

The RATE-AF Trial

RATE AF

Trial registration number: ISCRCTN 95259705

Statistical Analysis Plan

SAP Version Number
1.0

Author:	Samir Mehta	Role:	Trial Statistician	Affiliation:	BCTU
Signature:		Date:	16/12/2019		University of Birmingham
Reviewer:	Prof. Jon Deeks	Role:	Senior Statistician	Affiliation:	BCTU
Signature:		Date:	16/12/2019		University of Birmingham
Chief Investigator:	Dr. Dipak Kotecha	Role:	Chief Investigator	Affiliation:	Institute of Cardiovascular Sciences
Signature:		Date:	17/12/19.		University of Birmingham
**This Statistical Analysis Plan has been approved by:**					
Approver:		Role:	Senior Statistician	Affiliation:	BCTU
Signature:		Date:			University of Birmingham

Statistical Analysis Plan Amendments

SAP version number	Date Approved	Protocol version number†	Section number changed	Description of and reason for change	Timing of change with respect to interim/final analysis	Blind Reviewer	
						Name:	
						Signature:	
						Date:	
						Name:	
						Signature:	
						Date:	
						Name:	
						Signature:	
						Date:	

† This SAP was written based on information contained in the trial protocol version as listed here.

Abbreviations & Definitions	
**Abbreviation / Acronym**	**Meaning**
BCTU	Birmingham Clinical Trials Unit
CONSORT	Consolidated Standards of Reporting Trials
DMC	Data Monitoring Committee
ISRCTN	International Standard Randomised Controlled Trial Number
ITT	Intention to Treat
SAE	Serious Adverse Event
SAP	Statistical Analysis Plan
SUSAR	Suspected Unexpected Serious Adverse Reaction
TSC	Trial Steering Committee
QoL	Quality of life
PCS	Physical Component Score
MCS	Mental Component Score
**Term**	**Definition**
International Standard Randomised Controlled Trial Number	A clinical trial registry
Protocol	Document that details the rationale, objectives, design, methodology and statistical considerations of the study
Randomisation	The process of assigning trial subjects to intervention or control groups using an element of chance to determine the assignments in order to reduce bias.
Statistical Analysis Plan	Pre-specified statistical methodology documented for the trial, either in the protocol or in a separate document.

TABLE OF CONTENTS

1. Introduction 6
2. Background and rationale 6
3. Trial objectives 6
4. Trial methods 7
4.1. Trial design 7
4.2. Trial interventions 7
4.3. Primary outcome measure 7
4.4. Secondary outcome measures 7
4.5. Timing of outcome assessments 8
4.6. Randomisation 8
4.7. Sample size 8
4.8. Framework 8
4.9. Interim analyses and stopping guidance 8
4.10. Pilot Progression Rules 9
4.11. Timing of final analysis 9
4.12. Timing of other analyses 9
4.13. Trial comparisons 9
5. Statistical Principles 9
5.1. Confidence intervals and p-values 9
5.2. Adjustments for multiplicity 9
5.3. Analysis populations 10
5.4. Definition of adherence 10
5.5. Handing protocol deviations and violations 10
5.6. Unblinding 11
6. Trial population 11
6.1. Recruitment 11
6.2. Baseline characteristics 11
7. Intervention(s) 11
7.1. Description of the intervention(s) 11
7.2. Adherence to allocated intervention 11
8. Protocol deviations and violations 11
9. Analysis methods 11
9.1. Covariate adjustment 11
9.2. Distributional assumptions and outlying responses 12
9.3. Handling missing data 12
9.4. Data manipulations 12
9.5. Analysis methods – primary outcome(s) 18
9.6. Analysis methods – secondary outcomes 18
9.7. Analysis methods – exploratory outcomes and analyses 22
9.8. Safety data 22
9.9. Planned subgroup analyses 22
9.10. Sensitivity analyses 23
10. Analysis of sub-randomisations 23
11. Health economic analysis 23

12. **Statistical software** 23
13. **References** 24
Appendix A: Deviations from SAP 25
Appendix B: Trial schema 26
Appendix C: Schedule of assessments 27
Appendix D1: CONSORT flow diagram 28
Appendix D2: Baseline characteristics 29
Appendix D3: Adherence to allocated intervention 31
Appendix D4: Protocol deviations and violations 32
Appendix D5: Primary outcome results 33
Appendix D6: Secondary outcomes results 34
Appendix D7: Feasibility outcomes 41
Appendix D8: Safety 43
Appendix D9: Subgroup analysis for primary outcome 45

1. Introduction

This document is the Statistical Analysis Plan (SAP) for the RATE-AF trial, and should be read in conjunction with the current trial protocol. This SAP details the proposed analyses and presentation of the data for the main paper(s) reporting the results for the RATE-AF trial.

The results reported in these papers will follow the strategy set out here. Subsequent analyses of a more exploratory nature will not be bound by this strategy, though they are expected to follow the broad principles laid down here. The principles are not intended to curtail exploratory analysis (e.g. to decide cut-points for categorisation of continuous variables), nor to prohibit accepted practices (e.g. transformation of data prior to analysis), but they are intended to establish rules that will be followed, as closely as possible, when analysing and reporting data.

Any deviations from this SAP will be described and justified in the final report or publication of the trial (using a table as shown in Appendix A). The analysis will be carried out by an appropriately qualified statistician, who should ensure integrity of the data during their data cleaning processes.

2. Background and rationale

The background and rationale for the trial are outlined in detail in the protocol[1]. In brief, Atrial fibrillation (AF) is an increasingly common cardiac condition that leads to a substantial burden on quality-of-life (QoL), an increased risk of cardiovascular events, hospitalisation and death, and significant healthcare costs for the NHS. Beta-blocker monotherapy remains the first-line option in the current NICE AF guidelines consultation document, with digoxin only for sedentary patients, although this recommendation is based on very low-quality evidence.

The RAte control Therapy Evaluation in Atrial Fibrillation (RATE-AF) trial is Prospective, Randomised Open-label Blinded Endpoint (PROBE) clinical trial comparing the use of digoxin and beta-blockers as initial rate control therapy. The RATE-AF trial combines hypothesis testing (quality of life, cardiac function, exercise capacity and biomarkers), evaluation of measures (validity, reproducibility and correlation of outcomes) and a feasibility study for a future clinical event trial (assessing recruitment, retention and sample size).

3. Trial objectives

The primary objective is the patient-reported QoL, with a predefined focus on physical well-being using the SF-36v2 physical component summary at 6 months.

Secondary objectives are as follows:

- Generic and AF-specific patient-reported QoL using the SF-36 global and domain-specific scores, the AFEQT overall score and the EQ-5D-5L summary index and visual analogue scale at 6 and 12 months.
- Echocardiographic left-ventricular ejection fraction (LVEF) and diastolic function (E/e' and composite of diastolic indices) at 12 months.
- Functional assessment, including 6-minute walking distance achieved, change in European Heart Rhythm Association (EHRA) class and cognitive function at 6 and 12 months.
- Change in B-type natriuretic peptide (BNP) levels as a surrogate for total cardiac strain at 6 months.
- Change in heart rate from baseline and group comparison using 24-hour ambulatory ECG.

Feasibility objectives:

- Successful methods for recruitment
- Key issues that affect retention of participants, such as convenience, compliance and cross-over (target

of 85% study completion rate).

- Drug discontinuation rate and adverse reactions leading to drug discontinuation.
- Therapy-induced requirement for additional treatment (e.g. pacemaker implantation).
- Population-specific standard deviations and proportions to enable sample size calculation for a future trial.
- Assessment of cardiovascular outcomes including a composite of adverse clinical events (mortality, thromboembolic events, myocardial infarction and cardiovascular interventions).

4. Trial methods

4.1. Trial design

RATE-AF is a Prospective, Randomised Open-label Blinded Endpoint (PROBE) clinical trial comparing the use of Digoxin and beta-blockers (Bisoprolol) as initial rate control therapy. This study also designed to assess the feasibility of conducting a future clinical event trial. See Appendix B for trial schema.

4.2. Trial interventions

Digoxin 62.5-250 µg od
Bisoprolol 1.25-15 mg od

4.3. Primary outcome measure

The primary outcome is the Patient-reported Quality of life (QoL) SF-36v2 Physical Component Summary (PCS) score at 6 months.

4.4. Secondary outcome measures

Patient-reported QoL:

- SF-36 global and domain-specific scores at 6 and 12 months
- EQ-5D-5L summary index and visual analogue scale at 6 and 12 months
- AFEQT overall score at 6 and 12 months

Cardiac function:

- Echocardiographic LVEF at 12 months
- Diastolic function (E/e' and composite of diastolic indices) at 12 months
- Change in heart rate using 24-hour ambulatory ECG

Functional assessment:

- Six-minute walking distance at 6 and 12 months
- Change in European Heart Rhythm Association (EHRA) class at 6 and 12 months

Biomarkers:

- Change in B-type natriuretic peptide (BNP) levels at 6 and 12 months

Feasibility outcomes:

- Recruitment target of 3 patients per week across all participating centres
- Compliance and reasons for non-compliance
- Number of withdrawals and losses to follow-up (with reasons)
- Drug discontinuation rate and adverse reactions requiring drug discontinuation

- Number of patients needing therapy-induced requirement for additional treatment
- Cardiovascular events (mortality, thromboembolic events, myocardial infarction and cardiovascular intervention)
- Population-specific standard deviations (SD) and proportions for all outcomes

4.5. Timing of outcome assessments

The schedule of trial procedures and outcome assessments are given in Appendix C.

4.6. Randomisation

Participants will be randomised in a 1:1 ratio to either Digoxin 62.5 – 250 µg od or Bisoprolol 1.25 – 15 mg od. The time between randomisation and commencement of trial therapy should be minimised (ideally <24 hours).

Randomisation will be provided by a computer generated programme at the Birmingham Clinical Trials Unit (BCTU), using a minimisation algorithm incorporating the following factors:

- Baseline EHRA (class 1/2a and class 2b/3/4)
- Gender (Male and Female)

4.7. Sample size

Randomising 144 patients we can assume an 85% power to detect an effect size of half a standard deviation in a continuous outcome measure of QoL (two-sided alpha of 0.05).

A sample size of 160 patients would account for an estimated 10% loss to follow-up (including withdrawal and death prior to 12-month assessment).

There is some evidence from existing research to support the notion that the treatment effect could be this large. The mean SF-36 role-physical score from the rate-control arm of the RACE study was 47, with a 17% improvement with rate-control over time.[2] In another study, CCB resulted in 22% improvement in a proprietary symptom-checklist, compared to a non-significant 8% change in those assigned to beta-blockers (SD 10-points in both groups). These values are also consistent with a 17% improvement in SF-36 scores in a third trial, PIAF.[3]

4.8. Framework

The objective of the trial is to test the superiority of one intervention to another as well as to assess the feasibility of running a future clinical event study.

Null Hypothesis for primary outcome:
No difference in the SF-36v2 PCS score when comparing a strategy of digoxin versus beta-blocker therapy for initial rate control in patients with permanent AF.

Alternative Hypothesis:
Use of digoxin or beta-blocker therapy as initial rate control in patients with permanent AF is superior based on the PCS score from SF-36v2.

4.9. Interim analyses and stopping guidance

A joint oversight committee comprising a Trial Steering Committee (TSC) and Data Monitoring Committee (DMC) will be engaged for this trial. The role of the TSC is to provide the overall supervision of the trial. The TSC will

monitor trial progress and conduct and advice on scientific credibility. The TSC will consider and act, as appropriate, upon the recommendations of the Data Monitoring Committee. Further details of the remit and role of the TSC are available in the TSC Charter.

An independent DMC will be established to oversee the safety of participants in the trial. The DMC will meet prior to the trial opening to enrolment, and then meet at least annually, or as per a timetable agreed by the DMC prior to trial commencement. Data analyses will be supplied in confidence to the DMC, which will be asked to give advice on whether the accumulated data from the trial, together with the results from other relevant research, justifies the continuing recruitment of further participants. The DMC will operate in accordance with the trial specific charter. It is likely that the Haybittle-Peto boundary will be used. This states that if an interim analysis shows a probability of less than 0.001 that the treatments are different, then the trial should be stopped early. This will be used alongside data on important secondary endpoints and all other relevant evidence. A DMC report and charter outlining the terms of reference (including information on stopping rules) will be agreed with the DMEC.

4.10. Pilot Progression Rules

N/A

4.11. Timing of final analysis

The final analysis for the trial will occur after last randomised participant completes their 12-month follow-up and the corresponding outcome data has been entered onto the trial database and validated as being ready for analysis. This is provided that the trial has not been stopped early for any reason (e.g. DMC advice or funding body request).

4.12. Timing of other analyses

N/A

4.13. Trial comparisons

All references in this document to 'group' refer to Digoxin or Bisoprolol.

5. Statistical Principles

5.1. Confidence intervals and p-values

All estimates of differences between groups will be presented with two-sided 95% confidence intervals, unless otherwise stated. P-values will be reported from two-sided tests.

5.2. Adjustments for multiplicity

No correction for multiple testing will be made.

5.3. Analysis populations

All primary analyses (primary and secondary outcomes including safety outcomes) will be by intention-to-treat (ITT). Participants will be analysed in the intervention group to which they were randomised, and all participants shall be included whether or not they received the allocated intervention.

As a sensitivity analysis, a per-protocol analysis may also be carried out for the primary outcome if it is deemed a worthwhile investigation to further understand drug efficacy. See section 5.4 for how adherence information will be summarised. See section 9.10 for further details on any sensitivity analyses.

5.4. Definition of adherence

Data on adherence to medication was collected at each follow up visit and captured in two ways:

1. By asking the patients if they have taken "All", "Some" or "None" of their medication
   i) If patients have taken "Some" of their medication, then further asked if they have taken ">75%", ">50-75%", ">25-50%" or "≤25%"
2. By assessing the data on any oral medications that patient is taking to normalise their heart rate

Hence treatment adherence will be summarised in both ways described above and will be summarised separately for 6 and 12 months.

Per-Protocol population set:
Since the primary outcome for this study is at 6-months, the per-protocol population will therefore form of only those patients that have remained adherent to their treatment allocation at 6 months. Adherence to treatment allocation will be based on data collected on oral medication that the patients are taking at 6 months. Hence adherence will be computed as a binary "yes/no". The per-protocol set will therefore consist of patients that remained adherent to their treatment allocation at 6 months (based on data from oral medications) as well as those patients that remain in atrial fibrillation, as documented on the AFEQT questionnaire at 6 months.

Patients could also be taking additional rate control therapy beyond their randomised treatment allocation and so although not part of definition for adherence, this data will also be summarised by treatment arm. Similarly we also collect data on compliance by asking patients at each visit if they have been compliant with drugs used to control heart rate and so this data will also be summarised by treatment arm.

5.5. Handing protocol deviations and violations

A protocol deviation/violation is defined as a failure to adhere to the protocol such as errors in applying the inclusion/exclusion criteria, the incorrect intervention being given, incorrect data being collected or measured, follow-up visits outside the visit window or missed follow-up visits. We will apply a strict definition of the ITT principle and will include all participants as per the ITT population described in section 5.3 in the analysis in some form regardless of deviation from the protocol.[4] This includes participants who were randomised but later found to violate the inclusion or exclusion criteria. This does not include those participants who have specifically withdrawn consent for the use of their data in the first instance; however these outcomes will be explored as per other missing responses.

Where appropriate, additional sensitivity analysis for any protocol deviations and violations will be conducted for the primary outcome only. These will be described in section 9.10.

5.6. Unblinding

RATE-AF is an open label trial, blinded endpoint trial and so patients are unblinded however the investigators are blinded to the summary QoL scores at 6 and 12 months and detailed echocardiographic variables at 12 months. NTpro-BNP levels at 6 and 12 months are not known during the clinical consultation.

6. Trial population

6.1. Recruitment

A flow diagram (as recommended by CONSORT[5, 10]) will be produced to describe the participant flow through each stage of the trial. This will include information on the number (with reasons) of losses to follow-up (drop-outs and withdrawals) over the course of the trial. A template for reporting this is given in Appendix D1.

6.2. Baseline characteristics

The trial population will be tabulated as per Appendix D2. Categorical data will be summarised by number of participants, counts and percentages. Continuous data will be summarised by the number of participants, mean and standard deviation if deemed to be normally distributed or number of participants, median and interquartile range if data appear skewed, and ranges if appropriate. Tests of statistical significance will not be undertaken, nor confidence intervals presented.[6]

7. Intervention(s)

7.1. Description of the intervention(s)

N/A.

7.2. Adherence to allocated intervention

A cross-tabulation of allocated intervention by the adherence categories stated in section 5.4 will be produced (proportions and percentages). A template for reporting adherence is given in Appendix D3.

8. Protocol deviations and violations

Frequencies and percentages by group will be tabulated for the protocol deviations and violations as per Appendix D4.

9. Analysis methods

Intervention groups will be compared using appropriate statistical models, to adjust for all covariates as specified in section 9.1, where possible. See section 9.5 - 9.10 which describes in detail for each outcome the type of analysis method to be used.

9.1. Covariate adjustment

In the first instance, intervention effects between groups for all outcomes will be adjusted for the baseline score (where appropriate), minimisation parameters (Gender, baseline EHRA) as well as age at randomisation and

baseline LVEF (as continuous variables). The minimisation variable EHRA is a categorical score with the following categories (1, 2a, 2b, 3, 4) and for minimisation, this score was categorised into (class 1, 2a) and (class 2b, 3, 4). However for the analysis we will be adjusting this variable in its original 5 categorical form.

The Bisoprolol arm will be used as a reference category for all model based analyses.

For some binary outcomes, sometimes the effect size to be estimated of interest is the relative risk rather than the odds ratio and so for these outcomes, a log-binomial model is often used. However there are convergence issues with this type of model and so if the log-binomial model fails to converge, a Poisson regression model with robust standard errors will be used to estimate the same parameters.[7] If this also fails to converge, unadjusted estimates will be produced from the log-binomial model. It will be made clear in the final report why this occurred (e.g. not possible due to low event rate/lack of model convergence).

9.2. Distributional assumptions and outlying responses

Distributional assumptions (e.g. normality of regression residuals for continuous outcomes) will be assessed visually prior to analysis; although in the first instance the proposed primary method of estimation in this analysis plan will be followed. If responses are considered to be particularly skewed and/or distributional assumptions violated, the impact of this will be examined through sensitivity analysis; this will consist of transformation of responses prior to analysis (e.g. log transformation) in the first instance.

9.3. Handling missing data

In the first instance, analysis will be completed on received data only with every effort made to follow-up participants even after protocol violation to minimise any potential for bias. To examine the possible impact of missing data on the results, and to make sure we are complying with the intention-to-treat principle, sensitivity analysis will be performed on the primary outcome measure only.[8] See section 9.10 for further details.

9.4. Data manipulations

The Trial Statistician will derive all responses from the raw data recorded in the database:

Age at randomisation
(Randomisation date – Date of birth) / 365.25, taking the integer part of age

SF-36 version 2
<u>The SF-36v2 questions will be coded as follows</u>:

SFQ1 - Excellent=5
Very good=4.4
Good=3.4
Fair=2
Poor=1

SFQ2 - Much better now than one year ago = 5
Somewhat better now than one year ago = 4
About the same as one year ago = 3
Somewhat worse now than one year ago = 2
Much worse now than one year ago = 1

SFQ3a - Yes, limited a lot = 1
Yes, limited a little = 2

No, not limited at all = 3

SFQ3b - Yes, limited a lot = 1
Yes, limited a little = 2
No, not limited at all = 3

SFQ3c - Yes, limited a lot = 1
Yes, limited a little = 2
No, not limited at all = 3

SFQ3d - Yes, limited a lot = 1
Yes, limited a little = 2
No, not limited at all = 3

SFQ3e - Yes, limited a lot = 1
Yes, limited a little = 2
No, not limited at all = 3

SFQ3f - Yes, limited a lot = 1
Yes, limited a little = 2
No, not limited at all = 3

SFQ3g - Yes, limited a lot = 1
Yes, limited a little = 2
No, not limited at all = 3

SFQ3h - Yes, limited a lot = 1
Yes, limited a little = 2
No, not limited at all = 3

SFQ3i - Yes, limited a lot = 1
Yes, limited a little = 2
No, not limited at all = 3

SFQ3j - Yes, limited a lot = 1
Yes, limited a little = 2
No, not limited at all = 3

SFQ4a - All of the time = 1
Most of the time = 2
Some of the time = 3
A little of the time = 4
None of the time = 5

SFQ4b - All of the time = 1
Most of the time = 2
Some of the time = 3
A little of the time = 4
None of the time = 5

SFQ4c - All of the time = 1
Most of the time = 2
Some of the time = 3

A little of the time = 4
None of the time = 5

SFQ4d - All of the time = 1
Most of the time = 2
Some of the time = 3
A little of the time = 4
None of the time = 5

SFQ5a - All of the time = 1
Most of the time = 2
Some of the time = 3
A little of the time = 4
None of the time = 5

SFQ5b - All of the time = 1
Most of the time = 2
Some of the time = 3
A little of the time = 4
None of the time = 5

SFQ5c - All of the time = 1
Most of the time = 2
Some of the time = 3
A little of the time = 4
None of the time = 5

SFQ6 - Not at all = 5
Slightly = 4
Moderately = 3
Quite a bit = 2
Extremely = 1

SFQ7 - None = 6
Very mild = 5
Mild = 4
Moderate = 3
Severe = 2
Very severe = 1

SFQ8 - Not at all = 5
A little bit = 4
Moderately = 3
Quite a bit = 2
Extremely = 1

SFQ9a - All of the time = 5
Most of the time = 4
Some of the time = 3
A little of the time = 2
None of the time = 1

SFQ9b - All of the time = 1

Most of the time = 2
Some of the time = 3
A little of the time = 4
None of the time = 5

SFQ9c - All of the time = 1
Most of the time = 2
Some of the time = 3
A little of the time = 4
None of the time = 5

SFQ9d - All of the time = 5
Most of the time = 4
Some of the time = 3
A little of the time = 2
None of the time = 1

SFQ9e - All of the time = 5
Most of the time = 4
Some of the time = 3
A little of the time = 2
None of the time = 1

SFQ9f - All of the time = 1
Most of the time = 2
Some of the time = 3
A little of the time = 4
None of the time = 5

SFQ9g - All of the time = 1
Most of the time = 2
Some of the time = 3
A little of the time = 4
None of the time = 5

SFQ9h - All of the time = 5
Most of the time = 4
Some of the time = 3
A little of the time = 2
None of the time = 1

SFQ9i - All of the time = 1
Most of the time = 2
Some of the time = 3
A little of the time = 4
None of the time = 5

SFQ10 - All of the time = 1
Most of the time = 2
Some of the time = 3
A little of the time = 4
None of the time = 5

SFQ11a- Definitely true = 1
Mostly true = 2
Don't know = 3
Mostly false = 4
Definitely false = 5

SFQ11b- Definitely true = 5
Mostly true = 4
Don't know = 3
Mostly false = 2
Definitely false = 1

SFQ11c- Definitely true = 1
Mostly true = 2
Don't know = 3
Mostly false = 4
Definitely false = 5

SFQ11d- Definitely true = 5
Mostly true = 4
Don't know = 3
Mostly false = 2
Definitely false = 1

<u>The following domains will be computed from the SF-36 questionnaire</u>:

- Physical Function (PF) = SFQ3a + SFQ3b + SFQ3c + SFQ3d + SFQ3e + SFQ3f + SFQ3g + SFQ3h + SFQ3i + SFQ3j
- Physical Function Score = ((PF-10)/20)*100
- Role Limitation Due to Physical Problems (RP) = SFQ4a + SFQ4b + SFQ4c + SFQ4d
- Role Limitation Due to Physical Problems score = ((RP-4)/16)*100
- Role Limitation Due to Emotional Problems (RE) = SFQ5a + SFQ5b + SFQ5c
- Role Limitation Due to Emotional Problems Score = ((RE-3)/12)*100
- Social Functioning (SF) = SFQ6 + SFQ10
- Social Functioning Score = ((SF-2)/8)*100
- Mental Health (MH) = SFQ9b + SFQ9c + SFQ9d + SFQ9f + SFQ9h
- Mental Health Score = ((MH-5)/20)*100
- Energy/Vitality (EV) = SFQ9a + SFQ9e + SFQ9g + SFQ9i
- Energy/Vitality Score = ((EV-4)/16)*100
- Pain (P) = SFQ7 + SFQ8
- Pain Score = ((P-2)/9)*100
- General Health Perception (GHP) = SFQ1 + SFQ11a + SFQ11b + SFQ11c + SFQ11d
- General Health Perception Score = ((GHP-5)/20)*100

<u>AGPHYSCO</u>
(PF*0.456) + (RP*0.362) + (Pa*0.367) + (GHP*0.199) + (EV*-0.050) + (SF*-0.028) + (RE*-0.110) + (MH*-0.256)

<u>AGMENTCO</u>
(PF*-0.227) + (RP*-0.102) + (P*-0.130) + (GHP*0.036) + (EV*0.278) + (SF*0.272) + (RE*0.329) + (MH*0.460)

<u>Physical Component Summary score (PCS)</u>
(((AGPHYSCO-82.261)/20.867)*10)+50

Mental Component Summary score (MCS)
(((AGMENTCO-63.7796)/19.582)*10)+50

EQ-5D (5 level)
The current NICE guidelines (updated October 2019) on the use of EQ-5D-5L scoring based on the most recent value set for England published by Devlin et al. 2018 was not to use this and instead to map the 5L data into 3L value set based on mapping function developed by van Hout et al. 2012.

EQ-5D-5L have developed the crosswalk value sets for the 5L to 3L and so these values will be used for scoring: (https://euroqol.org/eq-5d-instruments/eq-5d-5l-about/valuation-standard-value-sets/crosswalk-index-value-calculator/).

For those patients that die prior to completing the EQ5D questionnaire, for the Index score a value of "0" will be imputed since for this questionnaire, a value of 0=death.

AFEQT questionnaire overall score

$$100 - \left[\frac{(\text{sum of severity for all questions answered} - \text{number of questions answered}) * 100}{\text{Total number of questions answered} * 6}\right]$$

*Note: ignore last two questions of the AFEQT questionnaire for scoring as they will be tabulated separately

IPAQ score (as continuous score)

- Sitting = N/A for IPAQ score
- Walking = 3.3 METs
- Moderate Intensity = 4.0 METs
- Vigorous Intensity = 8.0 METs

Total MET-minutes/week: MET level x minutes of activity/day x days per week
IPAQ score= Walk (3.3*min/day*days) + Moderate (4.0*min/day*days) + Vigorous (8.0*min/day*days)

NT-pro-BNP
Since this data is expected to be not normally distributed, a log transformation (natural log) for this data will need to be done to approximate normality prior to any analysis.

Composite of diastolic indices
This outcome will be coded as a binary yes/no, with "yes" representing patients that have a diastolic dysfunction present and "no" for patients that don't.

There is an algorithm to determine whether the patient has a diastolic function or not and it will be computed based on the following:

Does the patient have any **one** of the following diastolic parameters?

- Average E/e' (taken from lateral and septal wall) ≥ 15

    If yes, then Diastolic dysfunction present
    If no, then

Does the patient have **two** or more of the following diastolic parameters?

- IVRT (ms) ≤ 65 ms
- Mitral Valve E deceleration time (ms) ≤ 120ms
- Average E/e'(taken from lateral and septal wall) ≥ 11
- Pulmonary Vein diastolic deceleration time (ms) ≤ 220

    If yes, then Diastolic dysfunction present
    If no, then no diastolic dysfunction present

Change in European Heart Rhythm Association (EHRA) class
This outcome will be analysed as ordinal data initially but will also be analysed as a binary yes/no variable. The original classification for this score is in an ordinal scale and the categories are 1, 2a, 2b, 3, 4 where lowest category 1 indicates best outcome and highest category 4 indicates worst outcome. For this outcome to be coded as binary we will consider any one with a change in 2 categories from worse to better as "yes" for this outcome. Comparison will be made from baseline score to 6 months and baseline score to 12 months separately. EG: if a patient had a baseline EHRA class of 3a and by 6 months they had an EHRA class of 2a then this patient will be considered as "yes" for the classification of change in EHRA class. There may be some patients that cannot achieve a 2 point improvement in the score due to the score they originally had at baseline (i.e. if someone has a baseline score of 2a or below at baseline). These patients will be classed as not improved.

9.5. Analysis methods – primary outcome(s)

The primary outcome is the SF-36v2 physical component summary (PCS) score at 6 months.

The data for this outcome is continuous in nature and the computation for this score is described in the data manipulations section 9.4. The mean and standard deviation along with minimum and maximum values for the PCS score will be presented by treatment arm.

Data will be analysed using a linear regression model with outcome being the 6 months PCS score and independent variables in the model being the baseline PCS score, treatment arm, all minimisation variables, age at randomisation and baseline LVEF. An adjusted mean difference and 95% confidence interval will be estimated from the linear regression model and the p-value from the associated model will be produced. The Bisoprolol arm will be used as a reference category in the model and so higher values will indicate better outcome for Digoxin arm. A template for reporting the primary outcome is given in Appendix D5.

9.6. Analysis methods – secondary outcomes

A template for reporting all the secondary outcomes is given in Appendix D6.

Patient-reported QoL
For the RATE-AF trial, questionnaires SF-36v2, EQ-5D-5L and AFEQT are administered at baseline, 6 months and 12 months.

Up-titration visits:
These questionnaires are also administered for each patient at their last up-titration visit.
Note: the data for last up-titration visit is not done at any scheduled time-point due to the fact that each patient will have different up-titration visits (i.e. some will have 6 and some may only have 1) and so for this reason the data collected for last up-titration visit will only be summarised by treatment arm and no formal analysis for this data will be conducted.

- **SF-36v2 global and domain specific scores at 6 and 12 months**

The data for these outcomes are continuous in nature and the computation for the global and domain specific scores is described in the data manipulations section 9.4. The mean and standard deviation along with minimum and maximum values for global and domain specific scores will be presented by treatment arm and time-point.

The global and domain-specific scores will be analysed separately at 6 and 12 months using the same analysis methods as described in section 9.5. for primary outcome:
- Physical component summary (PCS) score at 12 months
- Mental component summary (MCS) score at 6 months and 12 months
- Physical Function score at 6 months and 12 months

- Role Limitation Due to Physical Problems score at 6 months and 12 months
- Role Limitation Due to Emotional Problems score at 6 months and 12 months
- Social Functioning score at 6 months and 12 months
- Mental Health score at 6 months and 12 months
- Energy/Vitality score at 6 months and 12 months
- Pain score at 6 months and 12 months
- General Health Perception score at 6 months and 12 months

The range for each domain of the SF-36v2 is from 0=worst score to 100=best score. The Bisoprolol arm will again be used as a reference category for all model based analysis for SF-36v2 and so higher values will indicate better outcome for Digoxin arm.

For SF36v2 PCS, additional secondary analysis will also be conducted using a mixed effects repeated measures model. The outcome in the model will be the repeated measure for PCS score and independent variables will be treatment arm, all minimisation variables, age at randomisation and baseline LVEF. Time (in days) will also be included in the model and a constant treatment effect over time will be assumed in the first instance, however a treatment by time interaction term will also be included in the model to check for its significance. If interaction is significant ($p<0.05$), then estimates at each time point will be produced from the model including the interaction term. An unstructured covariance data structure will be used in the model. Results will be presented as adjusted mean difference and 95% confidence interval.

- **EQ-5D-5L summary index and visual analogue scale at 6 and 12 months**

The data for these outcomes are continuous in nature and the computation for the index summary score is described in the data manipulations section 9.4. The visual analogue score (VAS) is obtained from a scale so this score doesn't need to be derived. The mean and standard deviation along with minimum and maximum values for index summary score and VAS score will be presented by treatment arm and time-point.

<u>The following will be analysed separately at 6 and 12 months using the same analysis methods as described in section 9.5. for primary outcome</u>:

- EQ-5D-5L summary index score at 6 months and 12 months
- EQ-5D-5L visual analogue scale score at 6 months and 12 months

The range for summary index is from -0.285=worst score to 1=best score and for visual analogue score is from 0=worst score to 100=best score. The Bisoprolol arm will again be used as a reference category for all model based analysis of EQ-5D-5L and so higher values will indicate better outcome for Digoxin arm.

- **AFEQT overall score at 6 and 12 months**

The data for this outcome is continuous in nature and the computation for the AFEQT overall score is described in the data manipulations section 9.4. The mean and standard deviation along with minimum and maximum values for AFEQT overall score will be presented by treatment arm and time-point.

<u>The following will be analysed separately at 6 and 12 months using the same analysis methods as described in section 9.5. for primary outcome</u>:

- AFEQT overall score at 6 months and 12 months

The range for AFEQT overall score is from 0=complete disability to 100=no disability. The Bisoprolol arm will again be used as a reference category for the model based analysis of this and so higher values will indicate better outcome for Digoxin arm.

Cardiac function

- **Echocardiographic LVEF at 12 months**

The data for this outcome is a continuous score (presented as a percentage of volume ejected) and is also categorised using the following categories; "<40%", "40-49%", "≥50%". This data is collected at baseline and at

12 months. This data will be summarised as the mean, standard deviation, minimum and maximum values for the continuous score as well as number and percentage for the categories by treatment arm and time-point.

The main analysis of this data will be based on the continuous data so this outcome will be analysed using the same analysis methods as described in section 9.5 for primary outcome. For this outcome only, additional covariates for history of myocardial infarction (MI) at baseline, coronary angioplasty or stents (PCI) at baseline and coronary artery bypass surgery (CAPG) at baseline will also be adjusted for in the model. Higher values of LVEF are considered better and since the Bisoprolol arm will again be used as a reference category for the model based analysis of this, higher values will indicate better outcome for Digoxin arm.

- **Diastolic function (E/e' and composite of diastolic indices) at 12 months**

The data for E/e' is a ratio and so continuous in nature. This data is collected at baseline and at 12 months. The mean and standard deviation along with minimum and maximum values will be presented by treatment arm and time-point.

This outcome will be analysed using the same analysis methods as described in section 9.5 for primary outcome. Lower values of E/e' are considered better and since the Bisoprolol arm will again be used as a reference category for the model based analysis of this, lower values will indicate better outcome for Digoxin arm.

The data for the composite of diastolic indices is a binary (yes/no) and will be computed as described in the data manipulations section 9.4. This data is collected at baseline and 12 months and will be summarised as number and percentage by treatment arm and time-point. The analysis for this outcome will be conducted using a logistic regression model, where the outcome will be the binary category (yes/no) at 12 months and independent variables in the model being the baseline category, treatment arm, all minimisation variables, age at randomisation and baseline LVEF. An adjusted odds ratio and 95% confidence interval will be estimated from the logistic regression model.

- **Change in heart rate**

The data type for heart rate is continuous in nature. This data is collected at baseline, 6 months and at 12 months. The mean and standard deviation along with minimum and maximum values will be presented by treatment arm and time-point for 1) Radial heart rate, 2) Apical heart rate, 3) 12-lead ECG heart rate, and 4) 24-hour ambulatory average heart rate. The 24-hour ambulatory heart rate is only measured once and so no baseline score will be there to adjust for it in analysis.

These outcomes will be analysed using the same analysis methods as described in section 9.5 for primary outcome and analysis will be done separately for 6 and 12 month time-points.

A scatter plot of radial vs apical heart rate at each time point will be produced to visualise the radial-apical discrepancy.

Functional assessment

- **Six-minute walking distance at 6 and 12 months**

The data for this outcome is continuous in nature and this test is conducted at baseline, 6 months and 12 months. The time (measured in min/s) and distance (measured in metres) are only recorded if the patient did the test. Therefore this data will be summarised by treatment arm and time-point with respect to the number of patients conducting the test, the mean and standard deviation, median and IQR as well as minimum and maximum values for time and distance covered. Reasons for stopping the test prematurely were also collected and so this will also be summarised by treatment arm and time-point.

The main endpoint for this outcome is the distance (in metres) walked and so this will be analysed using the same analysis methods as described in section 9.5 for primary outcome and analysis will be done separately for 6 and 12 month time-point.

- **Change in European Heart Rhythm Association (EHRA) class at 6 and 12 months**

The data for this outcome is categorical in nature and in 5 orderly categories; 1=None, 2a=Mild, 2b=Moderate, 3=Severe, 4=Disabling. This data is collected as baseline, 6 months and 12 months. This data will be summarised as number and percentage by treatment arm and time-point with respect to the EHRA class.

The analysis for this outcome will be conducted using an ordinal logistic regression model, where the outcome will be the EHRA class at follow up (with EHRA class 1 being the reference category) and independent variables in the model being the baseline EHRA class, treatment arm, gender, age at randomisation and baseline LVEF. An adjusted odds ratio and 95% confidence interval will be estimated from the ordinal logistic regression.

Higher EHRA class is considered to be a worse outcome and since the Bisoprolol arm will be used as a reference category in the model, higher values will indicate worse outcome for Digoxin arm.
Note: separate analysis will be done for 6 month and 12 month time-point.

We will also code this outcome as a binary "yes/no" variable where "yes" will be determined if patients had a 2 class improvement in the EHRA class from baseline. The full details for the computation of this are described in the data manipulations section 9.4.

The analysis for this outcome will be conducted using a logistic regression model, where the outcome will be the computed binary variable "yes/no" (with yes being the reference category) and independent variables in the model being treatment arm, gender, age at randomisation and baseline LVEF. An adjusted odds ratio and 95% confidence interval will be estimated from the logistic regression model.

Since we are modelling whether patients had an improvement from baseline and that the Bisoprolol arm will be used as a reference category in the model, higher values will indicate better outcome for Digoxin arm.
Note: again separate analysis will be done for 6 month and 12 month time-point.

Biomarkers

- **Change in NTpro-B-type natriuretic peptide (NTpro-BNP) levels**

The data for NTpro-BNP is continuous in nature and likely to be skewed and not normally distributed. Hence this data will need to be log-transformed first before analysis (see section 9.4 for more details). This data is collected at baseline, 6 month and 12 month. The raw untransformed data will be presented as mean standard deviation, median and interquartile range along with minimum and maximum values by treatment arm and time-point.

This outcome (log-transformed score) will be analysed using the same analysis methods as described in section 9.5 for primary outcome. Higher values of NTproBNP are considered worse and the Bisoprolol arm will again be used as a reference category for the model based analysis. Since we will be modelling the log-transformed scores and then exponentiate the effect size, the outcome will be in terms of geometric mean ratio and so values <1 will indicate better outcome for Digoxin arm.
Note: separate analysis will be done for 6 month and 12 month time-point.

Feasibility outcomes

- Recruitment target of 3 patients per week across all participating centres
- Compliance and reasons for non-compliance
- Number of withdrawals and losses to follow-up (with reasons)
- Drug discontinuation rate and adverse reactions requiring drug discontinuation
- Number of patients needing therapy-induced requirement for additional treatment
- Population-specific standard deviations (SD) and proportions:
    - SD of SF36 physical functioning score at 6 and 12 months
    - SD of SF36 overall score at 6 and 12 months

- SD of AFEQT overall score at 6 and 12 months
- SD of LVEF and E/e' scores at 6 and 12 months

- Unplanned hospitalisation admissions rates
- Cardiovascular Events (particularly mortality, thromboembolic events, myocardial infarction and cardiovascular interventions)

No formal model based analysis will be conducted for the feasibility outcomes and outcomes will be summarised using appropriate summary statistics.

A template for reporting this data is given in Appendix D7.

9.7. Analysis methods – exploratory outcomes and analyses

Any data that does not form a pre-specified outcome will be presented using simple summary statistics by intervention group (i.e. numbers and percentages for binary data and means (or medians) and standard deviations (or inter-quartile ranges) for continuous normal (or non-normal) data.

9.8. Safety data

The number and percentage of participants experiencing any adverse events, serious adverse events (SAEs) and suspected unexpected serious adverse reactions (SUSARs) will be presented by treatment arm. A table listing all the SAEs will be provided.

The safety data will also include summaries by treatment arm for:

- Digoxin levels at 6 and 12 months
- Number of patients requiring pacemaker implantation
- Unplanned hospitalisation rates (from the SAE form)
- Number of patients that had pauses and duration of pause (from the 24 hour tape form)
- All cardiovascular events (as recorded in the cardiovascular events form)
- Number of GP visits (from the GP form)

A template for reporting this data is given in Appendix D8.

9.9. Planned subgroup analyses

Interpretation of subgroup analysis will be treated with caution with output treated as hypothesis generating rather than definitive[9].

Analysis will be limited to the primary outcome and the following subgroups:

- Gender (Male, Female)
- Modified EHRA (Class 1/2a, Class 2b/3/4)
- Receiving beta-blocker therapy within 1 month of randomisation (No, Yes)
- Age (<75 years, ≥75 years)
- Left Ventricular Ejection Fraction (<50%, ≥50%)

The effects of these subgroups will be examined by including a treatment group by subgroups interaction parameter in the linear regression model.

A template for reporting is given in Appendix D9.

9.10. Sensitivity analyses

Sensitivity analyses will be limited to the primary outcome only and will consist of:

- Per-protocol analysis (population described in sections 5.3 and 5.4)
- Adjust the final model for additional covariate of baseline apical heart rate
- SF36v2 questionnaire completed outside the pre-specified visit window of ±4 week's

We have stated in the protocol that we will allow a ±4 week's window for the follow up visits and so any questionnaires for SF36v2 at 6 months completed outside this time window will be excluded in this sensitivity analysis.

- Analysis to assess the impact of missing data (see below for method)

Missing data will be imputed using multiple imputation with chained equations in Stata 16 (or above). Stata's "MI" command will be used to carry out this analysis and the "regress" option will be used since the primary outcome is continuous data. 50 imputations will be generated for any missing data for primary outcome (i.e. SF36v2 PCS score at 6 months) and all minimisation variables (Gender, EHRA score), treatment arm, baseline PCS score and any other baseline data deemed appropriate will be used to aid the multiple imputation procedure. Imputed results will be combined using Stata's "mi estimate" command.

10. Analysis of sub-randomisations

N/A

11. Health economic analysis

Health economic analysis is planned for this trial and will be described separately in the health economic analysis plan by the health economist for the trial.

12. Statistical software

Statistical analysis will be undertaken in the following statistical software packages:

- Stata version 15 (or higher)
- SAS software, version 9.4 (or higher)

13.References

1. Kotecha D, Calvert M, Deeks JJ, Griffith M, Kirchhof P, Lip GY, Mehta S, Slinn G, Stanbury M, Steeds RP, Townend JN. A review of rate control in atrial fibrillation, and the rationale and protocol for the RATE-AF trial. BMJ Open. 2017;7:e015099

2. Hagens VE, Ranchor AV, Van Sonderen E, Bosker HA, Kamp O, Tijssen JGP, Kingma JH, Crijns HJGM, Van Gelder IC. Effect of rate or rhythm control on quality of life in persistent atrial fibrillation: Results from the Rate Control Versus Electrical Cardioversion (RACE) study. J Am Coll Cardiol. 2004;43:241-247

3. Grönefeld GC, Lilienthal J, Kuck K-H, Hohnloser SH. Impact of rate versus rhythm control on quality of life in patients with persistent atrial fibrillation: Results from a prospective randomized study. Eur Heart J. 2003;24:1430-1436

4. Gupta SK. Intention-to-treat concept: A review. Perspect Clin Res. 2011;2(3):109-112.

5. Schulz KF, Altman DG, Moher D, for the CONSORT Group. CONSORT 2010 Statement: updated guidelines for reporting parallel group randomised trials. BMJ. 2010;340:c332.

6. Altman DG, Dore CJ. Randomisation and baseline comparisons in clinical trials. Lancet. 1990;335:149–53.

7. Zou G. A modified Poisson regression approach to prospective studies with binary data. Am J Epidemiol. 2004;159(7):702-6.

8. White IR, Horton NJ, Carpenter J, Pocock SJ. Strategy for intention to treat analysis in randomised trials with missing outcome data. BMJ. 2011;342:d40.

9. Wand R, Lagakos SW, Ware JH, Hunter DJ, Drazen JM. Reporting of subgroups analyses in clinical trials. NEJM. 2007;357:2189-94.

10. Melanie Calvert, PhD; Jane Blazeby, MD; Douglas G. Altman, DSc; et al; Reporting of Patient-Reported Outcomes in Randomized Trials: The CONSORT PRO Extension; JAMA. 2013;309(8):814-822. doi:10.1001/jama.2013.879

Appendix A: Deviations from SAP

This report below follows the statistical analysis plan dated <insert effective date of latest SAP> apart from following:

Section of report not following SAP	Reason

Appendix B: Trial schema

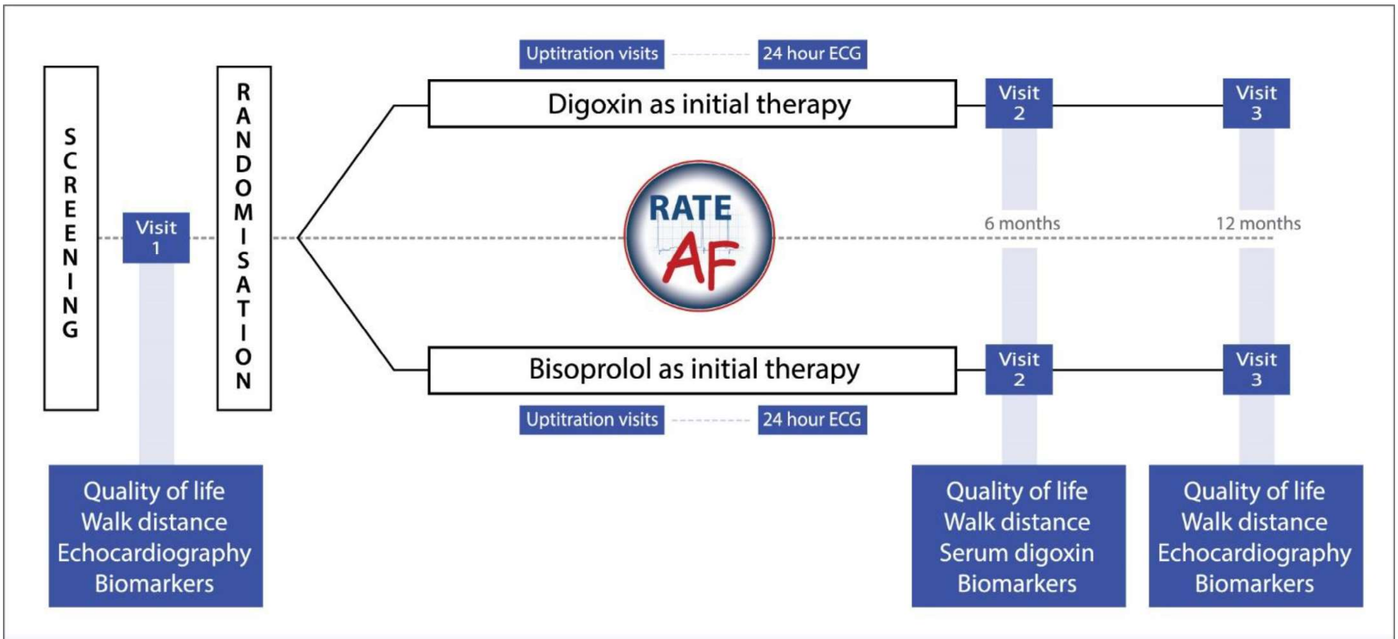

Inclusion Criteria:
- Age 60 years or older
- Permanent AF, characterised as a physician decision for rate control only
- Breathless; New York Heart Association Class II or above
- Able to provide written informed consent

Exclusion criteria:
- Established indication for beta-blocker therapy (e.g. myocardial infarction in the last 6 months)
- Known contraindication for beta-blockers or digoxin (e.g. history of severe bronchospasm or known intolerance)
- Baseline heart rate <60 bpm, 2nd/3rd degree block, accessory pathway or history of ventricular arrhythmia
- Planned pacemaker implantation, pacemaker-dependent rhythm or previous atrioventricular node ablation
- Decompensated heart failure within 14 days requiring intravenous inotropes, vasodilators or diuretics
- Hypertrophic cardiomyopathy, myo/pericarditis or previous/planned heart transplant
- Receiving renal replacement therapy
- Major surgery within 3 months or severe concomitant disease expected to reduce life expectancy

Appendix C: Schedule of assessments

Procedures		Baseline Visit	Up-titration Visits (Day 14 to 60)	Visit 2, Month 6 (± 4 weeks)	Visit 3, Month 12 (± 4 weeks)
Assessment of eligibility criteria		X			
Informed consent taken		X			
Review of medical history		X			
Review of medications		X	X	X	X
Physical exam	Complete	X			
	Symptom-directed		X	X	X
	Vital signs	X	X	X	X
Quality of life assessment		X	(X)	X	X
Functional and cognitive assessment		X		X	X
Transthoracic echocardiogram		X			X
12-lead electrocardiogram		X		X	X
6-minute walk test		X		X	X
24-hour ambulatory ECG			X	(X)	
Clinical labs	Chemistry	X		X	X
	Haematology	X		X	X
	Serum digoxin			(X)	(X)
Trial labs	BNP	X		X	
	Stored sample	X		X	
Assessment of compliance			X	X	X
Assessment of adverse events			X	X	X

Appendix D1: CONSORT flow diagram

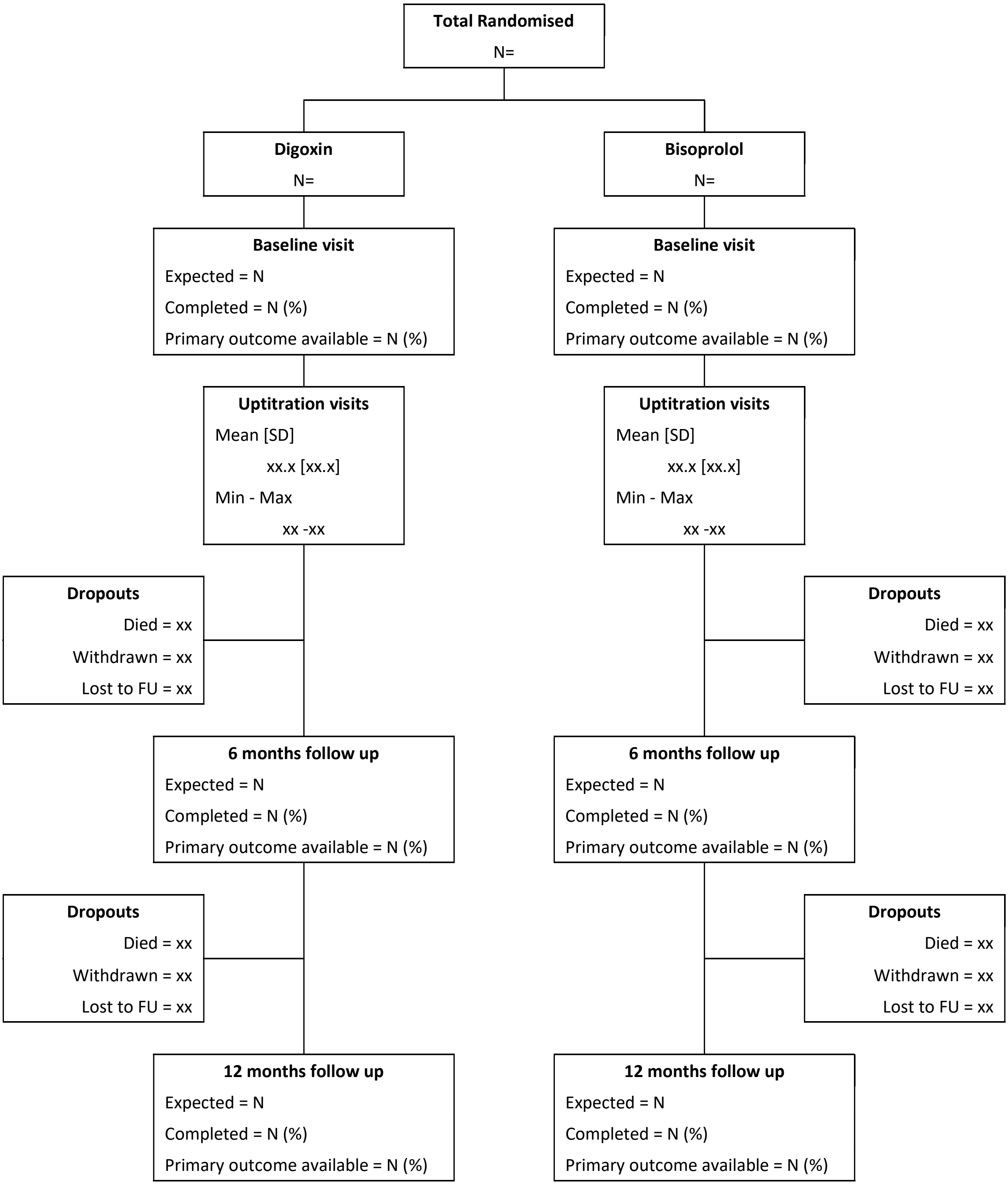

Appendix D2: Baseline characteristics

Baseline data		Digoxin (N=)	Bisoprolol	Total (N=)
**Age (Years)**				
	Mean [SD]			
	Med {IQR}			
	Min - Max			
**Gender***				
	Female			
	Male			
**Creatinine (micromol/L)**				
	Mean [SD]			
	Med {IQR}			
	Min - Max			
**On anticoagulant before randomisation**				
	No			
	Yes			
**EHRA class***				
	1			
	2a			
	2b			
	3			
	4			
**NYHA class**				
	I[£]			
	II			
	III			
	IV			
**Previous diagnosis of heart failure?**				
	No			
	Yes			
**Any signs of heart failure at baseline**				
	No			
	Yes			
**Type I diabetes**				
	No			
	Yes			
	missing			
**Type II diabetes**				
	No			
	Yes			
	missing			
**Unplanned admission for AF or HF in last 12 months**				
	No			
	Yes			
**Any previous cardioversions**				
	No			

Yes<br>Number of cardioversions (Min – Max)			
**Previously undergone AF ablation**<br>No<br>Yes<br>Number of ablations (Min – Max)			
**Previous history of anti-arrhythmic drugs**<br>No<br>Yes			
**Baseline NTproBNP (pg/mL)**<br>N<br>Mean [SD]<br>Med {IQR}<br>Min - Max			
**Radial artery heart rate (bpm)**<br>Mean [SD]<br>Med {IQR}<br>Min - Max			
**Apex beat heart rate (bpm)**<br>Mean [SD]<br>Med {IQR}<br>Min - Max			
**12-Lead ECG Heart Rate (bpm)**<br>N<br>Mean [SD]<br>Med {IQR}<br>Min - Max			
**Systolic BP (mmHg)**<br>N<br>Mean [SD]<br>Med {IQR}<br>Min - Max			
**Self-declared ethnicity**<br>White - English / Welsh / Scottish / Northern Irish / British<br>White - Irish<br>Asian / Asian British – Indian<br>Asian / Asian British – Pakistani<br>Black / African / Caribbean / Black British – Caribbean<br>Black / African / Caribbean / Black British – African			
**Estimated ejection fraction**<br>Mean [SD]<br>Median {IQR}<br>Min-Max<br><40%<br>40-49%<br>≥50%			

**Minimisation variables*
(Note: categories of the EHRA class for the minimisation algorithm were combined into EHRA class 1/2a and EHRA class 2b/3/4)

Appendix D3: Adherence to allocated intervention

Adherence to treatment allocation based on actual medication taken

Adherent to treatment allocation		Digoxin	Bisoprolol	Total
**At 6 months**				
	N			
	No			
	Yes			
If yes, taking additional rate control therapy				
	No			
	Yes			
**At 12 months**				
	N			
	No			
	Yes			
If yes, taking additional rate control therapy				
	No			
	Yes			

Oral medication type by treatment arm and time point

Medication*	6 months			12 months		
	Digoxin	Bisoprolol	Total	Digoxin	Bisoprolol	Total
Digoxin						
B-blocker						
Diltiazem						
Verapamil						
Amiodarone						
Other						

*Medications not mutually exclusive

Adherence assessed by asking the patient

Medication taken	6 months			12 Months		
	Digoxin (N=)	Bisoprolol (N=)	Total (N=)	Digoxin (N=)	Bisoprolol (N=)	Total (N=)
**All**						
**Some**						
>75%						
>50-75%						
>25-50%						
≤25%						
**None**						
**Missing**						

Appendix D4: Protocol deviations and violations

List of patients with follow-up visits conducted outside the specified ±4 week's window by treatment arm

Digoxin (N=xx)
1)
2)
…

Bisoprolol (N=xx)
1)
2)
…

Appendix D5: Primary outcome results

SF-36v2 physical component summary (PCS) score at 6 months

Primary outcome	Time point	Statistic	Digoxin	Bisoprolol	Linear regression model	
					Adjusted mean difference[1] 95% CI	P-value[1]
**Intention to treat analysis**						
**PCS**	**Baseline**	N<br>Mean [SD]<br>Min - Max			-	-
	**6 months**	N<br>Mean [SD]<br>Min - Max				
**Sensitivity Analysis – Multiple imputation for missing data**						
**PCS**	**Baseline**	N<br>Mean [SE]			-	-
	**6 months**	N<br>Mean [SE]				
**Per-Protocol analysis (i.e. adherent to treatment allocation and remained in permanent AF at 6 months)**						
**Included in per-protocol analysis set**	**6 months**	No<br>Yes			-	-
		**Total**				
**PCS**	**Baseline**	N<br>Mean [SD]<br>Min - Max			-	-
	**6 months**	N<br>Mean [SD]<br>Min - Max				

1-Adjusted for treatment arm, baseline score, all minimisation variables, age at randomisation and baseline LVEF
Reference group for the treatment arm in the model is Bisoprolol arm
Higher values indicate better scores so a positive mean difference favours Digoxin arm

Appendix D6: Secondary outcomes results

SF-36v2 global and domain specific scores at 6 and 12 months

Secondary outcome	Time point	Statistic	Digoxin	Bisoprolol	Linear regression model: Adjusted mean difference[1] 95% CI	Linear regression model: P-value[1]
**Physical Component Summary (PCS)**						
**PCS**	**Baseline**	N<br>Mean [SD]<br>Min - Max			-	-
	**12 months**	N<br>Mean [SD]<br>Min - Max				
**Mental Component Summary (MCS)**						
**MCS**	**Baseline**	N<br>Mean [SD]<br>Min - Max			-	-
	**6 months**	N<br>Mean [SD]<br>Min - Max				
	**12 months**	N<br>Mean [SD]<br>Min - Max				
**Physical Function Domain score (PF)**						
**PF**	**Baseline**	N<br>Mean [SD]<br>Min - Max			-	-
	**6 months**	N<br>Mean [SD]<br>Min - Max				
	**12 months**	N<br>Mean [SD]<br>Min - Max				
**Role Limitation Due to Physical Problems Domain score (RP)**						
**RP**	**Baseline**	N<br>Mean [SD]<br>Min - Max			-	-
	**6 months**	N<br>Mean [SD]<br>Min - Max				
	**12 months**	N<br>Mean [SD]<br>Min - Max				
**Role Limitation Due to Emotional Problems Domain score (RE)**						
**RE**	**Baseline**	N<br>Mean [SD]<br>Min - Max			-	-
	**6 months**	N<br>Mean [SD]<br>Min - Max				
	**12 months**	N<br>Mean [SD]<br>Min - Max				

**Social Functioning Domain score (SF)**						
**SF**	**Baseline**	N<br>Mean [SD]<br>Min - Max			-	-
	**6 months**	N<br>Mean [SD]<br>Min - Max				
	**12 months**	N<br>Mean [SD]<br>Min - Max				
**Mental Health Domain score (MH)**						
**MH**	**Baseline**	N<br>Mean [SD]<br>Min - Max			-	-
	**6 months**	N<br>Mean [SD]<br>Min - Max				
	**12 months**	N<br>Mean [SD]<br>Min - Max				
**Energy/Vitality Domain score (EV)**						
**EV**	**Baseline**	N<br>Mean [SD]<br>Min - Max			-	-
	**6 months**	N<br>Mean [SD]<br>Min - Max				
	**12 months**	N<br>Mean [SD]<br>Min - Max				
**Pain score (Pain)**						
**Pain**	**Baseline**	N<br>Mean [SD]<br>Min - Max			-	-
	**6 months**	N<br>Mean [SD]<br>Min - Max				
	**12 months**	N<br>Mean [SD]<br>Min - Max				
**General Health Perception Domain score (GHP)**						
**GHP**	**Baseline**	N<br>Mean [SD]<br>Min - Max			-	-
	**6 months**	N<br>Mean [SD]<br>Min - Max				
	**12 months**	N<br>Mean [SD]<br>Min - Max				

1-Adjusted for treatment arm, baseline score, all minimisation variables, age at randomisation and baseline LVEF
Reference group for the treatment arm in the model is Bisoprolol arm
Higher values indicate better scores so a positive mean difference favours Digoxin arm

EQ-5D-5L summary index and visual analogue scale at 6 and 12 months

Secondary outcome	Time point	Statistic	Digoxin	Bisoprolol	Linear regression model	
					Adjusted mean difference[1] 95% CI	P-value[1]
**EQ-5D-5L summary index score[£]**						
EQ-5D-5L summary index score	Baseline	N<br>Mean [SD]<br>Min - Max			-	-
	6 months	N<br>Mean [SD]<br>Min - Max				
	12 months	N<br>Mean [SD]<br>Min - Max				
**EQ-5D-5L visual analogue scale (VAS) score[$]**						
EQ-5D-5L VAS score	Baseline	N<br>Mean [SD]<br>Min - Max			-	-
	6 months	N<br>Mean [SD]<br>Min - Max				
	12 months	N<br>Mean [SD]<br>Min - Max				

1-Adjusted for treatment arm, baseline score, all minimisation variables, age at randomisation and baseline LVEF

£-The range for summary index is from -0.285=worst score to 1=best score

$-The range for visual analogue score is from 0=worst score to 100=best score

The Bisoprolol arm will be used as a reference category and so positive mean difference will indicate better outcome for Digoxin arm

AFEQT overall score at 6 and 12 months

Secondary outcome	Time point	Statistic	Digoxin	Bisoprolol	Linear regression model	
					Adjusted mean difference[1] 95% CI	P-value[1]
**AFEQT overall score[£]**						
AFEQT overall score	Baseline	N<br>Mean [SD]<br>Min - Max			-	-
	6 months	N<br>Mean [SD]<br>Min - Max				
	12 months	N<br>Mean [SD]<br>Min - Max				

1-Adjusted for treatment arm, baseline score, all minimisation variables, age at randomisation and baseline LVEF

£-The range for visual analogue score is from 0=worst score to 100=best score

The Bisoprolol arm will be used as a reference category and so positive mean difference will indicate better outcome for Digoxin arm

Echocardiographic LVEF at 12 months

Echocardiographic LVEF	Time point	Statistic	Digoxin	Bisoprolol	Linear regression model	
					Adjusted mean difference[1] 95% CI	P-value[1]
LVEF	Baseline	<40%<br>40-49%<br>≥50%			-	-
		N<br>Mean [SD]<br>Min - Max				
	12 months	<40%<br>40-49%<br>≥50%			-	-
		N<br>Mean [SD]<br>Min - Max				

1-Adjusted for treatment arm, baseline score, all minimisation variables, age at randomisation, baseline LVEF, history of myocardial infarction (MI) at baseline, coronary angioplasty or stents (PCI) at baseline and coronary artery bypass surgery (CAPG) at baseline
Reference group for the treatment arm in the model is Bisoprolol arm
Higher values indicate better scores so a positive mean difference favours Digoxin arm

Diastolic function (E/e' and composite of diastolic indices) at 12 months

Diastolic function	Time point	Statistic	Digoxin	Bisoprolol	Linear regression model	
					Adjusted mean difference[1] 95% CI	P-value[1]
E/e'	Baseline	N<br>Mean [SD]<br>Min - Max			-	-
	12 months	N<br>Mean [SD]<br>Min - Max				

1-Adjusted for treatment arm, baseline score, all minimisation variables, age at randomisation and baseline LVEF
Reference group for the treatment arm in the model is Bisoprolol arm
Higher values indicate better scores so a positive mean difference favours Digoxin arm

Diastolic function	Time point	Statistic	Digoxin	Bisoprolol	Logistic regression model	
					Adjusted Odds Ratio[1] 95% CI	P-value[1]
Composite of diastolic indices	Baseline	No<br>Yes			-	-
	12 months	No<br>Yes				

1-Adjusted for treatment arm, baseline score, all minimisation variables, age at randomisation and baseline LVEF
Reference group for the treatment arm in the model is Bisoprolol arm
Higher values indicate better scores so an odds ratio >1 favours Digoxin arm

Change in heart rate

Secondary outcome	Time point	Statistic	Digoxin	Bisoprolol	Linear regression model	
					Adjusted mean difference[1] 95% CI	P-value[1]
**Radial Heart (bpm)**						
**Radial Heart rate (bpm)**	**Baseline**	N<br>Mean [SD]<br>Min - Max			-	-
	**6 months**	N<br>Mean [SD]<br>Min - Max				
	**12 months**	N<br>Mean [SD]<br>Min - Max				
**Apical Heart rate (bpm)**						
**Apical Heart rate (bpm)**	**Baseline**	N<br>Mean [SD]<br>Min - Max			-	-
	**6 months**	N<br>Mean [SD]<br>Min - Max				
	**12 months**	N<br>Mean [SD]<br>Min - Max				
**12-lead ECG Heart rate (bpm)**						
**12-lead ECG Heart rate (bpm)**	**Baseline**	N<br>Mean [SD]<br>Min - Max			-	-
	**6 months**	N<br>Mean [SD]<br>Min - Max				
	**12 months**	N<br>Mean [SD]<br>Min - Max				
**24-hour ambulatory average Heart rate (bpm)**						
**24-hour ambulatory Heart rate**	**24-hour**	N<br>Mean [SD]<br>Min - Max				

1-Adjusted for treatment arm, baseline score, all minimisation variables, age at randomisation and baseline LVEF

The Bisoprolol arm will be used as a reference category

Six-minute walking distance at 6 and 12 months

Six-minute walking test	Digoxin (N=)	Bisoprolol (N=)	Total (N=)
**6 months follow-up**			
**Did the patient undergo the 6-min walk test?**			
No			
Yes			
Missing			
If yes:			
Total time spent (min/s)			
Median {IQR}			
Min - Max			
Total distance covered (m)			
Median {IQR}			
Min - Max			
Was the test stopped prematurely?			
No			
Yes			
**12 months follow-up**			
**Did the patient undergo the 6-min walk test?**			
No			
Yes			
Missing			
If yes:			
Total time spent (min/s)			
Mean [SD]			
Median {IQR}			
Min - Max			
Total distance covered (m)			
Mean [SD]			
Median {IQR}			
Min - Max			
Was the test stopped prematurely?			
No			
Yes			

Distance covered (in metres) from the 6-minute walk test

Secondary outcome	Time point	Statistic	Digoxin	Bisoprolol	Linear regression model	
					Adjusted mean difference[1] 95% CI	P-value[1]
Distance (metres)	Baseline	N<br>Mean [SD]<br>Min - Max			-	-
	6 months	N<br>Mean [SD]<br>Min - Max				
	12 months	N<br>Mean [SD]<br>Min - Max				

1-Adjusted for treatment arm, baseline score, all minimisation variables, age at randomisation and baseline LVEF

The Bisoprolol arm will be used as a reference category and so positive mean difference will indicate better outcome for Digoxin arm

Change in European Heart Rhythm Association (EHRA) class at 6 and 12 months

	Time point	Statistic	Digoxin	Bisoprolol	Ordinal Logistic regression model	
					Adjusted odds ratio[1]<br>95% CI	P-value[1]
EHRA Class	Baseline	Class 1<br>Class 2a<br>Class 2b<br>Class 3<br>Class 4			-	-
	6 months	Class 1<br>Class 2a<br>Class 2b<br>Class 3<br>Class 4				
	12 months	Class 1<br>Class 2a<br>Class 2b<br>Class 3<br>Class 4				

1-Adjusted for treatment arm, all minimisation variables, age at randomisation and baseline LVEF.
Reference group for the treatment arm in the model is Bisoprolol arm.
Lower odds indicate better outcome so values <1 favours Digoxin arm.

2 class improvement in EHRA class at 6 and 12 months compared to baseline

EHRA Class	Time point	Statistic	Digoxin	Bisoprolol	Logistic regression model	
					Adjusted Odds Ratio[1]<br>95% CI	P-value[1]
2 class improvement from baseline	6 months	No<br>Yes				
	12 months	No<br>Yes				

1-Adjusted for treatment arm, baseline score, all minimisation variables, age at randomisation and baseline LVEF
Reference group for the treatment arm in the model is Bisoprolol arm
Higher values indicate better scores so an odds ratio >1 favours Digoxin arm

Change in B-type natriuretic peptide (BNP) (NTproBNP) levels at 6 months

Secondary outcome	Time point	Statistic	Digoxin	Bisoprolol	Linear regression model	
					Ratio of geometric means [1]<br>95% CI	P-value[1]
Log-transformed NTproBNP (ng/L)	Baseline	N<br>Mean [SD]<br>Min - Max			-	-
	6 months	N<br>Mean [SD]<br>Min - Max				
	12 months	N<br>Mean [SD]<br>Min - Max				

1-Adjusted for treatment arm, baseline score, all minimisation variables, age at randomisation and baseline LVEF
Reference group for the treatment arm in the model is Bisoprolol arm
Lower ratio indicates better scores so values <1 favours Digoxin arm

Appendix D7: Feasibility outcomes

Recruitment target of 3 patients per week across all participating centres
To be presented graphically

Compliance and reasons for non-compliance
See section Appendix D3

Number of withdrawals and losses to follow-up (with reasons)

Drop-outs	Digoxin (N=)	Bisoprolol (N=)	Total (N=)
Lost to follow-up<br>Withdrawn<br>Death			
**Total**			

List of reason for withdrawals – Digoxin
1)
2)
3)
…

List of reason for withdrawals – Bisoprolol
1)
2)
3)
…

List of reason for death – Digoxin
1)
2)
3)
…

List of reason for death – Bisoprolol
1)
2)
3)
…

Drug discontinuation rate and adverse reactions requiring drug discontinuation

Has patient stopped medication due to AE's?	Digoxin (N=)	Bisoprolol (N=)	Total (N=)
No<br>Yes<br>Missing			
If yes, was it stopped:<br>Temporarily<br>Permanently			

Cardiovascular Events (from the cardiovascular event form and as identified from the SAE form)

- **List of all cardiovascular related events**

Number of patients needing therapy-induced requirement for additional treatment

Therapy-induced requirement	Digoxin (N=)	Bisoprolol (N=)	Total (N=)
**6 months follow-up**			
**Did the patient have a pacemaker fitted?**			
No			
Yes			
Missing			
If yes:			
Type of pacemaker			
Single chamber			
Dual chamber			
**12 months follow-up**			
**Did the patient have a pacemaker fitted?**			
No			
Yes			
Missing			
If yes:			
Type of pacemaker			
Single chamber			
Dual chamber			

Population-specific standard deviations (SD) and proportions

Outcome	6 months			12 months		
	Digoxin	Bisoprolol	Total	Digoxin	Bisoprolol	Total
**SF36 PCS**						
N - [SD]						
**SF36 MCS**						
N - [SD]						
**AFEQT**						
N - [SD]						
**LVEF**						
N - [SD]						
**E/e'**						
N - [SD]						

Unplanned hospitalisation rates (as recorded from the SAE form)

Unplanned hospitalisation rates	Digoxin (N=)	Bisoprolol (N=)	Total (N=)
No of patients exactly with:			
1 unplanned hospitalisation			
2 unplanned hospitalisation			
… unplanned hospitalisation			
**Total number of unplanned hospitalisation**			

Appendix D8: Safety

Adverse Events

Adverse event type	Digoxin		Bisoprolol		Total	
	N (%) of pts (N=)	*N of Events*	*N (%) of pts (N=)*	*N of Events*	*N (%) of pts (N=)*	*N of Events*
Gastrointestinal upset						
Blurred vision						
Rash						
Peripheral oedema						
Symptomatic bradycardia						
Dizziness						
Headache						
Lethargy						
Upper respiratory tract symptoms						
Symptomatic hypotension						
Other						
**Total**	**-**	**xx**	**-**	**xx**	**-**	**xx**
**N of pts with at least one AE**	**xx (xx%)**		**xx (xx%)**		**xx (xx%)**	
**Chi^2 test for difference in number of patients with at least one AE between treatment groups P-value = x.xxx**						

SAE's

SAE's	Digoxin (N=)	Bisoprolol (N=)	Total (N=)	Chi^2 Test P-value
Patients with at least one SAE:				
No				
Yes				
No of patients exactly with:				
1 SAE				-
2 SAE's				-
… SAE's				-
**Total number of SAE's**				-

- **List of all SAE's**

GP visits by trial arm at 6 and 12 months

GP visits	Digoxin (N=)	Bisoprolol (N=)	Total (N=)
**6 months follow-up**			
**Has the patient seen their GP since last trial visit?**			
No			
Yes			
Missing			
If yes, how many times:			
N			
Mean [SD]			
Median {IQR}			
Min-Max			
**Total number of visits for all patients**			
**12 months follow-up**			
**Has the patient seen their GP since last trial visit?**			
No			
Yes			
Missing			
If yes, how many times:			
N			
Mean [SD]			
Median {IQR}			
Min-Max			
**Total number of visits for all patients**			

Number of patients that had pauses and duration of pause (from the 24 hour tape form)

Pauses and duration from 24-hour tape	Digoxin (N=)	Bisoprolol (N=)	Total (N=)
**Did the patient have any pauses?**			
No			
Yes			
Missing			
If yes, maximal pause duration (seconds):			
N			
Mean [SD]			
Median {IQR}			
Min-Max			

Digoxin levels at 6 and 12 months for Digoxin arm

Digoxin levels (ug/l)	6 months	12 months
N		
Mean [SD]		
Median {IQR}		
Min-Max		

Appendix D9: Subgroup analysis for primary outcome

Subgroup analysis for PCS of SF36v2 at 6 months

Subgroup	Adjusted Mean difference (95% CI)	Interaction P-value
**Gender**		
Male		
Female		
**Modified EHRA class**		
(1, 2a)		
(2b, 3, 4)		
**Receiving beta-blocker therapy within 1 month of randomisation**		
No		
Yes		
**Age (in years)**		
<75 years		
≥75 years		
**Left Ventricular Ejection Fraction (%)**		
<50%		
≥50%		

Forest plot of subgroup analysis for primary outcome

UNIVERSITY OF BIRMINGHAM | INSTITUTE OF CARDIOVASCULAR SCIENCES | RATE AF | cardAIc

Addendum: RATE-AF wearables substudy

Joint Statistical Analysis Plan (SAP) and Artificial intelligence Analysis Plan (AiAP)

Chief Investigator:	Prof. Dipak Kotecha	Role:	Chief Investigator	Affiliation:	Institute of Cardiovascular Sciences University of Birmingham
Signature:	[signature]	Date:	22/7/2021		

Analysis to follow the same principles/intention-to-treat approach as noted in the RATE-AF trial SAP (version 1.0).

Addendum for RATE-AF wearables substudy to section 4.7 – Sample size:

As an exploratory analysis, no sample size calculation will be performed in advance of recruitment. To evaluate the number of sub-study participants required, a sample size calculation will be performed using 5 weeks of heart rate sensor data on the first 10 patients, providing average weekly heart rate, SD and correlation of repeated measures.

Addendum for RATE-AF wearables substudy to section 4.8 – Framework:

The wearable sensor sub-study contributes to the prespecified secondary outcome in the RATE-AF trial of change in heart rate comparing digoxin vs beta-blockers (null hypothesis: no difference in heart rate when comparing a strategy of digoxin versus beta-blocker therapy for initial rate control in patients with permanent AF).
The wearable sensor neural network approach contributes to the prespecified secondary outcomes in the RATE-AF trial of change in functional classification and 6-minute walking distance (null hypothesis: no difference between sensor-derived continuous variables and single time-point measurement of 6-minute walking distance/ECG heart rate in relation to change in New York Heart Association [NYHA] functional class; note change from QoL outcome).

Addendum for RATE-AF wearables substudy to section 9.4 – Data manipulations:

Sensor data will be treated as a continuous two-channel time series, pooled at 1-minute intervals and standardised: heart rate scaled to have zero mean and unit variance, and step count normalised to interval [0,1] to preserve the zero property of measurements. A third channel denoting data missingness will be added to capture missing data dependencies without imputation.

Addendum for RATE-AF wearables substudy to section 9.7 – Analysis methods – exploratory outcomes:

Conventional statistics: The analysis of heart rate will occur over a 20-week period of collected sensor data for each participant after reaching steady-state of randomised treatment allocation. The regression approach will use generalised estimating equation models, with random-effects to account for multiple repeated measurements. Adjusted models will account for age (continuous), gender (female/male), diagnosis of heart failure (yes/no) and NT-pro B-type natriuretic peptide (continuous). A specific regression model will account for any interaction with physical activity levels as determined by corresponding sensor data step counts.
Machine learning: Sensor data from a one-week period at the end of the trial will be held as a validation set. The remaining data will be augmented to train an unsupervised convolutional neural network (CNN) on a discrimination task. The augmentation will create a secondary dataset with heart rate/step count channels randomly permuted across patients. The unsupervised CNN will be trained to discriminate between real and permuted data, learning the relationship (non-linear) between sensor channels. The specifics of the convolutional architecture (depth, width, connectivity etc.) are hyper-parameters that will be determined for the appropriate model. Training samples will include staggered 4-hour windows of sensor data. After training, the model will be applied to the window preceding assessment of NYHA class using normalised logistic regression models. F1 scores will be calculated with 95% CI estimated by bootstrap resampling for: (1) the sensor data; (2) clinical factors (age, gender and body mass index); and (3) measurements taken at the periodic trial visits (ECG heart rate and 6-minute walk test).

Addendum for RATE-AF wearables substudy to section 12 – Statistical software:

Machine learning analyses will be performed using Python and TensorFlow (latest versions).
