# Supplementary material for: Consumer wearable devices for evaluation of heart rate control using digoxin versus beta-blockers: the RATE-AF randomized trial
Source: Nat Med. 2024 Jul 15;30(7):2030–6. doi: 10.1038/s41591-024-03094-4 (PMC11271403; doi:10.1038/s41591-024-03094-4)
Supplement: Supplementary file 2 — Reporting Summary [file 41591_2024_3094_MOESM2_ESM.pdf]

nature portfolio

Corresponding author(s): Dipak Kotecha

Last updated by author(s): May 13, 2024

Reporting Summary

Nature Portfolio wishes to improve the reproducibility of the work that we publish. This form provides structure for consistency and transparency in reporting. For further information on Nature Portfolio policies, see our Editorial Policies and the Editorial Policy Checklist.

Statistics

For all statistical analyses, confirm that the following items are present in the figure legend, table legend, main text, or Methods section.

n/a	Confirmed	
☐	☒	The exact sample size (*n*) for each experimental group/condition, given as a discrete number and unit of measurement
☐	☒	A statement on whether measurements were taken from distinct samples or whether the same sample was measured repeatedly
☐	☒	The statistical test(s) used AND whether they are one- or two-sided<br>*Only common tests should be described solely by name; describe more complex techniques in the Methods section.*
☐	☒	A description of all covariates tested
☐	☒	A description of any assumptions or corrections, such as tests of normality and adjustment for multiple comparisons
☐	☒	A full description of the statistical parameters including central tendency (e.g. means) or other basic estimates (e.g. regression coefficient) AND variation (e.g. standard deviation) or associated estimates of uncertainty (e.g. confidence intervals)
☐	☒	For null hypothesis testing, the test statistic (e.g. *F*, *t*, *r*) with confidence intervals, effect sizes, degrees of freedom and *P* value noted<br>*Give P values as exact values whenever suitable.*
☒	☐	For Bayesian analysis, information on the choice of priors and Markov chain Monte Carlo settings
☒	☐	For hierarchical and complex designs, identification of the appropriate level for tests and full reporting of outcomes
☐	☒	Estimates of effect sizes (e.g. Cohen's *d*, Pearson's *r*), indicating how they were calculated

Our web collection on statistics for biologists contains articles on many of the points above.

Software and code

Policy information about availability of computer code

Data collection	Data collection was automated using the RADAR-base platform, funded by the European Union Innovative Medicines Initiative RADAR-CNS (115902). This platform allows for secure streaming of data from wearables, apps and devices.
Data analysis	Machine learning analyses were performed with custom coding developed using Python (Python Software Foundation, Delaware; version: continually updated) with scikit-learn, and TensorFlow (Google Brain, California). The machine learning framework for embedding multi-channel time-series data is made freely available at: https://github.com/gkoutos-group/wearable_data_embedding. Statistical analyses were performed using Stata version 17 (StataCorp LP, Texas), with a two-tailed p-value <0.05 denoting statistical significance.

For manuscripts utilizing custom algorithms or software that are central to the research but not yet described in published literature, software must be made available to editors and reviewers. We strongly encourage code deposition in a community repository (e.g. GitHub). See the Nature Portfolio guidelines for submitting code & software for further information.

Data

Policy information about availability of data

All manuscripts must include a data availability statement. This statement should provide the following information, where applicable:

- Accession codes, unique identifiers, or web links for publicly available datasets
- A description of any restrictions on data availability
- For clinical datasets or third party data, please ensure that the statement adheres to our policy

Summary anonymised wearable sensor data are available for non-commercial purposes on request to the corresponding author (Prof Dipak Kotecha; d.kotecha@bham.ac.uk; 60-days response time for decisions). Due to the risk of patient re-identification, access to any individual-level data will require an appropriate ethical committee approval and review by the RATE-AF trial oversight committee which includes patient and public representatives (applications to Prof Dipak Kotecha; d.kotecha@bham.ac.uk; 180-days response time for decisions). Anonymised RATE-AF main trial and substudy datasets will be made available in an open-access repository after completion of secondary manuscripts.

Research involving human participants, their data, or biological material

Policy information about studies with human participants or human data. See also policy information about sex, gender (identity/presentation), and sexual orientation and race, ethnicity and racism.

Reporting on sex and gender	Gender used as this was self-identified by participants on enrollment.<br>Gender reported in main results and table 1. Gender accounted for in adjusted analyses. Added gender interaction for main result.
Reporting on race, ethnicity, or other socially relevant groupings	Ethnicity was self-reported by participants on enrollment, as required by UK policies on diversity and equality for research within the National Health Service. The UK census categories were used for ethnicity reporting.<br>Ethnicity is reported in the limitations as this was primarily a White British/Irish population. Full data on ethnicity have already been published (JAMA 2020;324:2497-2508).
Population characteristics	Fifty-three participants were enrolled in the sub-study, with mean age at randomisation of 75.6 years (SD 8.4; range 61 to 90 years) and 40% women. 28 (53%) had been randomised to digoxin and 25 (47%) to beta-blockers a mean of 30 weeks prior to their entry into the wearables sub-study (SD 8 weeks; range 12 to 46 weeks). Both groups were well balanced with respect to demographics and clinical measurements, with the commonest comorbidities being hypertension (74%) and heart failure (45%).
Recruitment	Recruitment for the RATE-AF trial occurred from 3 hospitals and primary care sites in the West Midlands, England, between 2016 and 2018. Inclusion criteria were: (1) age 60 years or older; (2) permanent AF in need of rate-control, with no plans to restore sinus rhythm; and (3) symptoms of possible heart failure, with breathlessness equivalent to New York Heart Association (NYHA) class II or above. Exclusion criteria were limited so the population enrolled reflected clinical practice. Patients that met these selection criteria were invited to join the trial (see JAMA 2020;324:2497-2508 for full details). Funding for the wearables sub-study was obtained after the main trial had commenced from the European Union Innovative Medicines Initiative BigData@Heart programme (116074). Participants with at least 2 months remaining in the RATE-AF trial were considered eligible for inclusion in the sub-study. All eligible participants were invited to join the wearable substudy regardless of age, gender, clinical status or familiarity with technology. 53 patients joined the study and 19 declined (see Extended Data Table 1 for characteristics).
Ethics oversight	Ethical approval was obtained from the East Midlands–Derby Research Ethics Committee (16/EM/ 0178), the Health Research Authority (IRAS 191437), and the Medicines and Healthcare Products Regulatory Agency.

Note that full information on the approval of the study protocol must also be provided in the manuscript.

Field-specific reporting

Please select the one below that is the best fit for your research. If you are not sure, read the appropriate sections before making your selection.

☒ Life sciences ☐ Behavioural & social sciences ☐ Ecological, evolutionary & environmental sciences

For a reference copy of the document with all sections, see nature.com/documents/nr-reporting-summary-flat.pdf

Life sciences study design

All studies must disclose on these points even when the disclosure is negative.

Sample size	As an exploratory analysis, no sample size calculation was performed in advance of recruitment. Sensor data from the first 5 weeks of heart rate data in the first 10 participants provided average weekly heart rate, SD and correlation of repeated measures, indicating that a sample size of 40 participants would provide 90% power over 20 weeks (2-sided alpha 0.05) to detect a 1/3 SD difference in heart rate (2 beats/min) between digoxin and beta-blockers (values inserted from the data extraction on first 10 participants: control 72 beats/min; SD 6 beats/min; repeated measures correlation 0.9). A minimum target of 50 enrolled participants was targeted to account for death and loss to follow-up during the substudy.

Data exclusions	One patient from each arm was lost to follow up, with 51 participants included in the final analysis.
Replication	A post-hoc subgroup analysis according to activity levels was based on US Centers for Disease Control recommended activity levels (minimum 150 minutes/week aerobic activity equivalent to 15,000 steps/week, and health benefits goal of 300 minutes/week aerobic activity equivalent to 30,000 steps/week).<br>CNN F1 scores used bootstrapping. See methods for full details on evaluation of machine learning approaches.<br>The wearable sensor data neural network was retrained by a different data scientist with generation of new code to test the validity of our original model. The results for the wearables neural network prediction were extremely stable and demonstrated successful replication:<br>Scientist#1: F1 score for NYHA class prediction=0.55 (95% CI 0.40 - 0.70)<br>Scientist#2: F1 score for NYHA class prediction=0.56 (95% CI 0.41 - 0.70).
Randomization	As part of the main RATE-AF trial, each participant was randomised to either digoxin 62.5-250µg or bisoprolol 1.25-10mg once daily (or alternative beta-blockers) in a 1:1 ratio. Randomisation was completed using a computer-generated minimisation algorithm to ensure treatment arms were balanced for gender and AF symptoms, based on the modified European Heart Rhythm Association classification score.
Blinding	The RAte control Therapy Evaluation in permanent Atrial Fibrillation (RATE-AF) trial was a prospective, randomised, open-label trial embedded in usual care.

Reporting for specific materials, systems and methods

We require information from authors about some types of materials, experimental systems and methods used in many studies. Here, indicate whether each material, system or method listed is relevant to your study. If you are not sure if a list item applies to your research, read the appropriate section before selecting a response.

Materials & experimental systems

n/a	Involved in the study
☒	☐ Antibodies
☒	☐ Eukaryotic cell lines
☒	☐ Palaeontology and archaeology
☒	☐ Animals and other organisms
☐	☒ Clinical data
☒	☐ Dual use research of concern
☒	☐ Plants

Methods

n/a	Involved in the study
☒	☐ ChIP-seq
☒	☐ Flow cytometry
☒	☐ MRI-based neuroimaging

Clinical data

Policy information about clinical studies
All manuscripts should comply with the ICMJE guidelines for publication of clinical research and a completed CONSORT checklist must be included with all submissions.

Clinical trial registration	registered with clinicaltrials.gov (NCT02391337) and clinicaltrialsregister.eu (2015-005043-13)
Study protocol	available at JAMA 2020;324:2497-2508
Data collection	Recruitment occurred from 3 hospitals and primary care sites in the West Midlands, England, between 2016 and 2018. Substudy sensor data collection between 2018 and 2019.
Outcomes	Endpoints acquired in the main trial were patient-reported quality of life, N-terminal pro-hormone B-natriuretic peptide, symptoms and functional capacity using mEHRA and NYHA class, 6-minute walk distance and time, heart rate (pulse examination), 12-lead ECG, left ventricular ejection fraction using cardiac ultrasound, and assessment of adverse events. The primary and secondary outcomes were predefined in the protocol (see JAMA 2020;324:2497-2508).<br>Substudy endpoints for the wearable sensor data were focused on heart rate and step count (as detailed in the pre-specified addendum to the RATE-AF trial statistical analysis plan) to test the null hypothesis of no difference between sensor-derived continuous variables and single time-point assessment using 6-minute walk distance and electrocardiograph heart rate for predicting change in New York Heart Association functional class from substudy start to trial end. Wearable sensor data were pooled at 1-minute intervals to form time-series data, with an additional channel to denote missingness.

Plants

Seed stocks	N/A
Novel plant genotypes	N/A
Authentication	N/A
